# Defining order and timing of mutations during cancer progression: the TO-DAG probabilistic graphical model

**DOI:** 10.3389/fgene.2015.00309

**Published:** 2015-10-13

**Authors:** Paola Lecca, Nicola Casiraghi, Francesca Demichelis

**Affiliations:** ^1^Laboratory of Computational Oncology, Centre for Integrative Biology, University of TrentoTrento, Italy; ^2^Department of Physiology and Biophysics, Institute for Computational Biomedicine, Weill Medical College of Cornell UniversityNew York, NY, USA

**Keywords:** mutagenetic graphs, timed graphs, cumulative cancer progression models, graph inference, prostate cancer, melanoma

## Abstract

Somatic mutations arise and accumulate both during tumor genesis and progression. However, the order in which mutations occur is an open question and the inference of the temporal ordering at the gene level could potentially impact on patient treatment. Thus, exploiting recent observations suggesting that the occurrence of mutations is a non-memoryless process, we developed a computational approach to infer timed oncogenetic directed acyclic graphs (TO-DAGs) from human tumor mutation data. Such graphs represent the path and the waiting times of alterations during tumor evolution. The probability of occurrence of each alteration in a path is the probability that the alteration occurs when all alterations prior to it have occurred. The waiting time between an alteration and the subsequent is modeled as a stochastic function of the conditional probability of the event given the occurrence of the previous one. TO-DAG performances have been evaluated both on synthetic data and on somatic non-silent mutations from prostate cancer and melanoma patients and then compared with those of current well-established approaches. TO-DAG shows high performance scores on synthetic data and recognizes mutations in gatekeeper tumor suppressor genes as trigger for several downstream mutational events in the human tumor data.

## Introduction

The systematic analyses of human tumor genomes in the last decade revealed that cancer is due to the combined effect of multiple driver mutations that accumulate and impair cell growth, cell division, and cell destruction (Vogelstein et al., 1988; Stratton et al., 2009; Greaves and Maley, 2012; Garraway and Lander, 2013). Many oncogenes and tumor suppressor genes contributing to tumorigenesis when activated or inactivated by acquired mutations have been identified, but the order in which those deleterious mutations occur is poorly understood (Futreal et al., 2004; Michor et al., 2004; Merlo et al., 2006; Gerstung et al., 2011; Sun et al., 2014). The sequence in which genetic events occur is of fundamental interest to understand carcinogenesis, progression and ultimately to guide therapeutics (Weinstein and Joe, 2008; Cheng et al., 2012; Turajlic et al., 2015). Specifically, causal models of cancer progression based on *timed graphs* (dependency graphs representing timed processes/events connected by causal dependency relationships) of genomic alterations can identify those mutations that induce oncogenic addiction and may thus represent promising therapeutic targets (Cheng et al., 2012).

The inference of timed graphs from cross- sectional data is a challenging task. First, the order and the timing of the genetic alterations can vary among patients (inter-patient heterogeneity) and even among different tumor nodules from the same patient (intra-patient heterogeneity; Vogelstein et al., 1988; Gerlinger et al., 2012; Alizadeh et al., 2015; Beltran and Demichelis, 2015). Second, the process of accumulating mutations is more complex than what can be represented by a single path (Cheng et al., 2012). Building on the pioneering linear model proposed by Vogelstein et al. (1988), statistical methods considering branch like trees and graphs have been proposed (Radmacher et al., 2001; Beerenwinkel et al., 2005; Rahnenführer et al., 2005; Attolini et al., 2010; Gerstung et al., 2011; Cheng et al., 2012; Longerich et al., 2012). In a recent comprehensive review (Hainke et al., 2012), the methods are grouped in: (i) oncogenetic tree models (oncotrees; Desper et al., 2000; Beerenwinkel et al., 2005), (ii) Bayesian graphical approaches (Radmacher et al., 2001; Gerstung et al., 2009, 2011; Sakoparnig and Beerenwinkel, 2012), and (iii) approaches based on clustering and evolutionary fitting algorithms (Michor et al., 2004; Attolini et al., 2010; Cheng et al., 2012). These methods generate graphs where the nodes are genetic events (at the level of single genes or at the level of gene pathways) and the directed arcs between nodes denote a relationship between them. Figure S1 shows examples of trees and graphs of different complexity obtained from the co-occurrence tables (Figure S1A) with probabilistic tree-based approaches (oncotree, Figure S1B), Bayesian inference (Figure S1C), evolutionary algorithms (Figure S1D), and the probabilistic graph modeling approach we developed (Figure S1E).

Briefly, the directed tree structures of oncotrees represent the probabilities of accumulating further mutations along divergent temporal sequences; each mutation can be represented only once and can have multiple subsequent mutations (child mutations) independently occurring; edges weights that are transition probabilities from the parent mutation to the child mutation. Distance-based oncotree approach involves generating a phylogenetic tree over all events using a distance measure between mutational events, where leaf nodes represent the set of possible events (Desper et al., 2000). Further development of cancer progression modeling by oncotree is known as mixture tree model and includes multiple oncogenetic trees, each of which can independently lead to cancer development. An expectation maximization algorithm is then used to determine the most likely tree mixture to fit the data (Beerenwinkel et al., 2007a). One acknowledged restriction of tree-based methods is that they preclude the possibility of converging evolutionary paths that occur when multiple alterations result in the same phenotypic effect. Furthermore, they impose a strict ordering of events. Bayesian graphical methods on the other hand can include converging evolutionary paths (Hjelm et al., 2006; Gerstung et al., 2009). For instance Conjunctive Bayesian Networks (CBNs) allow for multiple parental nodes thereby modeling the synergistic effects of multiple events in promoting subsequent mutations and describe the accumulation of events that are constrained in the order of their occurrence (Beerenwinkel et al., 2007b). The continuous time CBN (CT-CBN; Gerstung et al., 2011) also includes an explicit timeline, making quantitative predictions about the waiting time of mutations and, consequently about the speed of the tumor progression (Hainke et al., 2012). Detailed descriptions of the three categories of methods are presented in Supplementary Material.

The network-like representation of temporal order and the relationships between genetic events provided by probabilistic graph models and evolutionary fitting algorithms made their use much more widespread than for oncotrees. However, the theoretical model of the majority of graphs is based on pairwise dependencies between genetic events; it assumes that mutations are random events and that the accumulation process is a memoryless stochastic process. Based on these assumptions, current graph models are a simplistic generalization of the tree models and allow only the specification of stochastic process having the Markov property. Such memoryless property holds when the conditional probability distribution of the process next states depends only on the current state and not on the sequence of states that preceded it. While current graph models paved the way to a network-based modeling of the order and timing of mutation events, new theoretical frameworks for new computational models of probabilistic graphs need to be developed to overcome these limitations.

Overcoming the Markov property assumption and the limitation to “pairwise” dependencies between genetic events might allow for causal dependency inference that better resembles the real mutation accumulation process dynamics of cancer formation. In this direction, we developed a novel probabilistic graph model named Timed Oncogenetic Directed Acyclic Graph (TO-DAG) aimed at estimating the order and the waiting time of mutation events. Different than previously proposed methods, TO-DAG doesn't use Markov chains, but defines a conditional probability measure on the mutational patterns state space, for which the occurrence of a mutation in a pattern is conditionally dependent on the occurrence of all the preceding mutations. Such probabilities can be estimated from the occurrence data of a set of genetic mutations in a tumor/patient sample. Paths of mutations having non-null statistically significant probabilities define the topological structure of the cancer progression model. The waiting times of the mutation events are estimated a posteriori as inversely proportional to their conditional probability. In this way TO-DAG inference method decouples the prediction of the interdependencies among mutations from the estimate of the exact time of occurrence of these mutations. Namely, the conditional probability of a mutation is not defined as an explicit function of the exact time of occurrence and determines only the presence/absence of edges in a path of mutations. This probability is then interpreted as the rate of the occurrence of a mutation and used to estimate the waiting time elapsing from the occurrence of a mutation and its successor.

We first applied TO-DAG both on randomly generated synthetic data and on structured non-random synthetic data (i.e., generated in a controlled manner) to test its performance with respect to the number of genes, the number of samples and the events frequencies and to assess to what extent the output graphs reflect the input data structure. Next we turned to genetic data that we recently generated from 74 human prostate cancer samples that include point mutations, copy number losses and gains, and rearrangements (Barbieri et al., 2012; Baca et al., 2013). The models generated by TO-DAG have been extensively compared with the trees and the graphs inferred by most recent tools representative of the three classes, Oncogenetic tree (Oncotree; Szabo and Boucher, 2002), CT-CBN (Gerstung et al., 2011), Retracing the Evolutionary Steps in Cancer (RESIC; Attolini et al., 2010).

The following sections of the manuscript present our novel methodology, the mutation networks inferred from synthetic and real data and the discussion in the light of extensive comparison with the networks inferred by the other methods.

## Methods: timed oncogenetic directed acyclic graph (TO-DAG)

TO-DAG is an inferential method that deduces from cross-sectional data of genetic alterations in tumor patients the causal dependencies and the waiting times among these genetic events. From matrices with genetic events and patient samples as rows and columns, respectively, TO-DAG generates a probabilistic graph model whose nodes represent genetic events and oriented edges between nodes indicate the presence and the direction of a causal dependency between the nodes. A direct acyclic graph, i.e., a graph with no directed cycles, has been specifically chosen as model of putative causal dependencies, as genetic alterations are assumed to be irreversible events. Two parameters define an edge: (i) its probability estimated from the frequency of occurrence of the genetic events represented by the nodes and its conditional probability, and (ii) the waiting time, i.e., the time elapsing from the occurrence of a mutation to the occurrence of another one that is conditionally dependent on it.

Six main steps define the inferential procedure of TO-DAG:

1. Calculation of the probability of genetic event mutation. The probability of an event is calculated as the frequency of the event in sample set.
2. Calculation of the conditional probability of each pair of genetic events and construction of a complete directed graph whose edge weights are proportional to the conditional probabilities of the occurrence of the genetic events represented by the target nodes, given that the genetic event of the source nodes has occurred.
3. Elimination of edges with low probability.
4. Estimation of the waiting time between two events as realization of an exponential process with rate proportional to the conditional probability of the child event given the parent event.
5. Update of the probability of each genetic event with the probability of the event conditional to the occurrence of its predecessors.
6. Graph path reduction to eliminate low-probability mutation paths.

In the following we will describe in details the formalization of the graph model generated by TO-DAG. Adopting the notation from Szabo and Boucher (2002) to describe TO-DAG algorithm:

- let *n* be the number of genetic events
- let *p*_*i*_ be the probability that the *i*-th genetic alteration occurs, and *i* = 1, 2, …, *n*
- let *p*_*i*∪*j*_ be the probability that the *i*-th or the *j*-th or both the genetic alterations occur, and *i, j* = 1, 2, …, *n*
- let *p*_*i*∩*j*_ be the probability that both the *i*-th and the *j*-th alteration occur, and *i, j* = 1, 2, …, *n* provided that *i* ≠ *j*
- Let *p*_*i*|*j*_ be the probability that the *i*-th genetic event occurs given that the *j*-th alteration has occurred. By the definition of conditional probability *p*_*i*|*j*_ is
  $$\begin{array}{l} p_{i|j}=\frac{p_{i\cap j}}{p_{j}},\;i,j=1,2,\;\ldots ,\;n;i\neq j \end{array}$$

The steps of the oncogenetic graph reconstruction algorithm are defined as follow.

1. Estimate *p*_*i*_ and *p*_*i*∩*j*_, *i, j* = 1, 2, …, *n* (*i* ≠ *j*) from the data using the above definitions.
2. Construct a complete DAG on vertices {*v*_1_, *v*_2_, …, *v*_*n*_} representing the occurrence of the individual events conditioned to their predecessor, with edge weights defined as
  $$\begin{array}{l} w\left(v_{i},v_{j}\right)\equiv \;\frac{p_{j|i}}{p_{i}+p_{j}}\;=\frac{p_{i\cap j}}{p_{i}\left(p_{i}+p_{j}\right)} \end{array}$$

for an edge from vertex *v*_*i*_ to a vertex *v*_*j*_.

Accordingly to these definitions, an edge between two vertices is drawn if the conditional probability of the target vertex given the source vertex is not null. Weights are then assigned to the edges *v*_*j*_ → *v*_*i*_ for which *p*_*i*|*j*_ is large compared to the individual probabilities *p*_*i*_ and *p*_*j*_. The direction of an edge between node *i* and node *j* is directed from *i* to *j* if *w*(*v*_*i*_, *v*_*j*_) > *w*(*v*_*j*_, *v*_*i*_) and from *j* to *i* if *w*(*v*_*i*_, *v*_*j*_) < *w*(*v*_*j*_, *v*_*i*_). If *w*(*v*_*i*_, *v*_*j*_) = *w*(*v*_*j*_, *v*_*i*_) an undirected edge between *i* and *j* is drawn. In the present implementation of the algorithm, no threshold is set on the tolerance within which to compare the equality of the edge weights. Based on this definition of edge weight and this rule for determining the edge orientation, and edge between *i* and *j* is oriented from *i* to *j* if *p*_*i*_ > *p*_*j*_, i.e., from the most frequently mutated gene to the less frequently mutated gene.

3. For a given confidence level γ, for all the edges in the graph (i.e., edges with positive weight), define a confidence interval for *p* ≡ *p*_*i*_ + *p*_*j*_. Assuming that the sample mean of the mutation probability *p* is normally distributed with sampling variability given by *p*(1 − *p*)∕*n*, the confidence interval is delimited by the roots of the polynomial
  $$\begin{array}{l} F_{i|j}\left(p\right)={\left[p_{i|j}-p\right]}^{2}-z_{\gamma }\leq 0\forall i,j=1,2,\ldots ,n \end{array}$$

where *p*_*i*|*j*_ is estimated from the data, and *z*_γ_ is the quantile of order γ or the standard Normal distribution.

Let *p*_*min*_ and *p*_*max*_ be the roots of the polynomial *p*_*i*|*j*_(*p*) remove from the graph the edges for which

$$\begin{array}{l} p_{j|i}<p_{i}+p_{j}+\left(p_{max}-p_{min}\right) \end{array}$$

This step implements a comparison between the nominator and the denominator in the expression of *w*(*v*_*i*_, *v*_*j*_) to determine an interval on *p*_*i*_ + *p*_*j*_ beyond which we can consider the conditional probability *p*_*j*|*i*_ significantly greater than the sum of the single probabilities.

Store the conditional probabilities *p*_*j*|*i*_ satisfying this condition.

4. For each event *k* in a pathway connecting *k* to its *n*_*k*_ predecessors (and for each pathway in the graph), update the probability of the edge of the vertices (*v*_*k* − 1_, *v*_*k*_) in the following way
  $$\begin{array}{l} p_{k|k-1}\leftarrow p_{k|\left(k-1,k-2,\ldots ,k-n_{k}\right)}=p_{k\cap \left(k-1\right)\cap \left(k-2\right)\cdots \cap \left(k-n_{k}\right)} \end{array}$$

(see Figure S10).

This step implements the replacement of the conditional probability of event *k* given its direct predecessor *k* − 1 with the probability of the event *k* conditional to all the predecessor of event *k*. Remove from the graph those paths for which the following condition is satisfied

$$\begin{array}{l} p_{k\cap \left(k-1\right)\cap \left(k-2\right)\cdots \cap \left(k-n_{k}\right)}<\overset{n_{k}}{\underset{h=0}{\sum }}p_{k-h|k-\left(h+1\right)} \end{array}$$

5. For each edges *v*_*i*_ → *v*_*j*_ draw a realization △*t* from an exponential distribution
  $$\begin{array}{l} \Phi \;\sim \lambda \exp\left(-\lambda △t\right),\;\lambda \equiv p_{i|j} \end{array}$$

where △*t* is the waiting time, and the rate parameter λ is just the conditional probability of the event *j* conditional to its predecessor *i*, we stored at step 3. According to this definition, the waiting time, i.e., the time elapsing from a mutation to a subsequent one, is a random variable simulated by as a Markov process. Thus, the waiting times are random and independent each from the others, to reflect the impossibility to infer the exact time of occurrence of a mutation from the observed data.

We assume that the Markovian property holds only for the sequence of waiting times, and is not used to calculate the event probabilities that define the edge distribution. Therefore, Markovian processes do not define the topology of the causal dependency graph. Figure 11S schematizes the assignment of waiting time to the graph edges.

6. Waiting time based graph clustering to define three categories: “fast” edges (△*t* < *t*_1_), “moderate” edges (*t*_1_ ≤ △*t* ≤ *t*_2_), and “slow” edge (△*t* > *t*_2_), where *t*_1_ and *t*_2_ are the first and the third quartiles of the waiting times distribution.

In the next section, we will present TO-DAG results obtained on synthetic and real data and the comparison with other methods. We refer the reader to Supplementary Material for a detailed description of the three recent methods selected for the comparison, Oncotree (Szabo and Boucher, 2002), CT-CBN (Gerstung et al., 2011), and RESIC (Attolini et al., 2010), representative of the three main classes of methods for determining causal and temporal topologies of mutation pathways.

## Results and discussion: TO-DAG performances and comparative analysis with other methods

Table 1 summarizes the characteristics of the considered methods such as the type of output (tree or graph, timed network or non-temporal model), the assumptions (e.g., Markovian property of the mutation accumulation process), and technical features such as the size of the input co-occurrence tables. Here we report and compare the networks inferred with TO-DAG and with Oncotree, CT-CBN, and RESIC considering two sets of input data: (i) random uncorrelated data of different sizes generated by varying the number of mutation events or/and the number of samples, and (ii) synthetic data generated by changing the mutation frequency of each genetic event (i.e., the percentage of samples affected by a genetic aberration) or by introducing disjoint sets of samples (i.e., sets of samples that do no share any mutation event). The first set of experiments aims at measuring the maximum size of the input co-occurrence table in terms of number of events and number of mutational events, the computational complexity, and the number of predicted edges as a function of the input size. The latter is considered as a measure of false positives. The second set of experiments tests the extent to which different approaches are able to reflect the data structure in the inferred graphical models.

**Table 1:** **Output format, properties of inference models and performances of Oncotree, CT-CBN, RESIC, and TO-DAG**.

	**Oncotrees**	**CT-CBN**	**RESIC**	**TO-DAG**
Graph		X	X	X
Timed network		X	X	X
Topology/conditional probability tables	X			X
Non-memoryless				X
**PERFORMANCES**				
Max nr. of Mutation events	~1K (^*^)	29	~1K	~10K
Max nr. of Samples	~0.1K (^*^)	~100	~1K	~10K

(

* *) Oncotree-package admits input matrices of larger dimensions however, the correct ordering of estimated probabilities of the events and the computation of the joint probabilities cannot be addressed with limited sample sizes and computational power*.

### Experiments on random binary input matrices

The input to Oncotree, CT-CBN and TO-DAG is a matrix with samples (S1, S2,…, S20) in rows and mutation events/genes (E1, E2,…, E20) in columns, whereas for RESIC is the transposed matrix (see Figure S2A). Random binary matrices with an equal number of positive entries and null entries have been generated through the Walker's random sampling method by imposing a mutation frequency of 50% for each genetic event (Breitung, 1989).

#### Oncotrees

One hundred oncotrees have been inferred from 100 co-occurrence matrices reporting a number of mutation events/genes increasing from 10 to 1000 by 10 and a number of samples equal to 50. The increment of the number of inferred edges vs. the number of genes resulted to be linear (Figure S2A). Similarly, 100 oncotrees have been inferred from 100 co-occurrence matrices reporting a number of samples increasing from 10 to 1000 by 10 and a number of genes equal to 50. The number of edges vs. the number of samples resulted to be a stiff negative exponential zeroing at approximately 200 genes (Figure S2D). The addition of samples to a binary matrix with random uncorrelated entries makes the number of putative edges converge to zero, i.e., the larger the number of observations on a random binary process is, the smaller is the number of predicted relationships among the instances of the process.

Figures S3A–D show the average edge weight and the variances of the edge weights with respect to the number of genes (Figures S3A,B) and the number of samples (Figures S3C,D).

#### CT-CBN

CT-CBN was tested on smaller co-occurrences tables as the method can handle 30 samples maximum. Number of edges vs. number of genes has been obtained on six co-occurrence matrices with genes increasing from 5 to 30 by incremental step of 5 and number of samples equal to 7. Similarly, for the number of arcs vs. the number of samples, CT-CBN was applied to six matrices with sample sizes increasing from 5 to 50 by incremental step of 5 and number of genes equal to 7. Although the modest size of the input matrix, the inferential processing for a mutation frequency of the 50% was computationally intractable with this method.

The increment of the number of arcs vs. the number of genes is linear as with the oncotree method (Figure S2B), whereas the decrement of the number of arcs associated to the increment of the number of samples follows a hyperbolic behavior (Figure S2E).

In CT-CBN the edge weights are calculated through bootstrapping in multiple runs. However, given the small size of the input matrix that this approach can process, estimates of average edge weight and variance of the average edges weights are not considered of statistical significance and thus are here omitted.

#### RESIC

RESIC does not infer edges from uncorrelated random data suggesting that false positives rates are likely low.

#### TO-DAG

TO-DAG infers a number of edges quadratically increasing with the number of genes (see Figure S2C for 50 samples) and following a sigmoidal function with respect to the number of samples (see Figure S2F for 50 genes). Sample size increment does not lead to lower number of putative edges as for oncotree and CT-CBN methods, rather it stabilizes on a plateau. This behavior is expected for models that maintain/track memory of the past states. Furthermore, the level of the plateau of the sigmoid obtained on random input can be interpreted as a measure of false positive edges inferred by the method from real data, provided that the random input matrix shows the same mutation frequency of the real data. The existence of an analytical relationship between the probability of a path in the graph and the height of the sigmoid should be further assessed and potentially used to determine a threshold to control for false positives.

Figures S3E–H show the behavior of the average edge weight and the variances of the edge weights vs. number of genes (Figures S3E,F) and vs. number of samples (Figures S3G,H). Finally, we analyzed the rate parameter λ of the exponential distribution as function of the number of genes and number of samples. Figure S4A indicates an oscillatory behavior of λ when the number of genes is less than 100 (and the number of samples is fixed to 50), then followed by a more stable one when the number of genes increases. Of note is the curve reported in Figure S4B that indicates that λ decreases as the number of samples increases till the null value. This suggests that if the number of samples is significantly higher than the number of genes, the distribution of the waiting times can be approximated without loss of accuracy to a uniform distribution, and the waiting time of mutation can be calculated as the inverse of λ.

We also explored two topological indices (assortativity and transitivity) and the exponential rate of the waiting time distribution against an increment of the mutation frequency to evaluate and validate the performance of TO-DAG in inferring the structure of interconnections among mutations. In this experiment, mutation frequency changes have been realized by randomly assigning different percentages of positive values to the columns (samples) of the co-occurrence table. The results are reported in Figure S5. Assortativity is here operationalized as the correlation between the total degree (i.e., number of edges) of two nodes. Transitivity (also known as clustering coefficient) measures the probability that the adjacent vertices of a vertex are connected. As the mutation frequency increases we observed (i) a decrement of the assortativity as (Figure S5A); (ii) a hump-shaped behavior of the median of the distribution of local transitivity (Figure S5B); and (iii) a linear increase of the parameter of the waiting time distribution. Results (i) and (ii) indicate a weakening of the relationship between nodes as the mutation frequency increases, whereas result (iii) is a trivial consequence (by definition of “rate” of a random Poisson process) of the augment of the frequency of mutation.

While the strength of the relationship decreases as the mutation frequency increases, we correspondingly observe a moderate increment of the average edge weight and a moderate decrease of the variance of distribution of edge weights (see Figure S6).

### Experiments on controlled binary input matrices

Synthetic data were generated both with a step-wise increase of the mutation frequency and by forcing disjoint sample sets, i.e., sets of samples that have no genetic event in common.

#### Step-wise increment of mutation frequency

**Figure 1:**
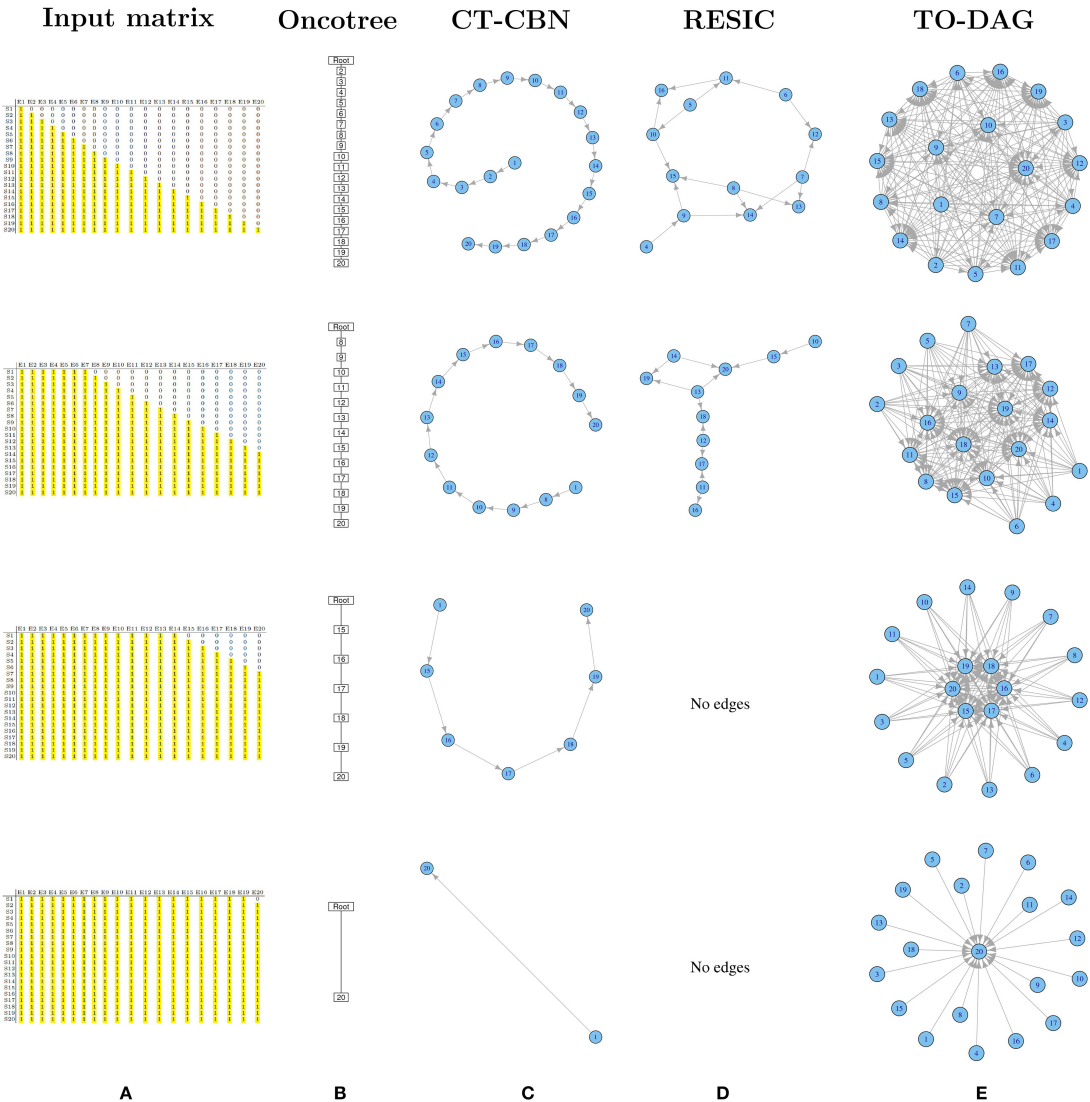
**Output graphs for synthetic data generated by step-wise increment of mutation rates**. **(A)** Input matrices and corresponding **(B)** Oncotree tree graphs, **(C)** graphs inferred by CT–CBN method, **(D)** graphs inferred by RESIC algorithm, and **(E)** graphs inferred by TO–DAG.

A set of binary 20 by 20 matrices was generated starting from a binary triangular matrix and step-wise incrementing the mutation frequencies of genes. Figure 1A illustrates the step-wise increment: the triangular matrix is the starting configuration; at each step the other matrices are obtained by progressively converting the first 0 of each row in a positive entry until the matrix is filled with 1s. The following pseudo-code explains how the matrices are generated starting from a binary lower triangular matrix *A*(*n* × *n*) = {*x*_*i, j*_}. At each step the new matrix obtained with this procedure is saved and used as input to TO-DAG.

*step*: = 0
  while(*step* < *n* − 1)
         {
           for(*i in* 1 : *n* − 1 − *step*)
              {
                *x*(*i, j*+1 − *step*) ← 1;
                *A_step_* ← *A*{ *x* } ;
                save {*A_step_*} ;
                }
             *step* ← *step* + 1
           }

For each matrix obtained with this procedure, the graph of the mutation events has been inferred with the four approaches.

#### Oncotree

Figure 1B shows that as the frequency of mutation increases the number of events separated from the root decreases. Columns entirely filled by 1s are not separable from the root event. In the extreme case in which all except the last column are entirely filled by 1s, the tree shows the root connected with the twentieth event.

#### CT-CBN

CT-CBN output is identical to the output of the oncotree method (see Figure 1C). Importantly, as CT-CBN implementation only considers mutation events that are distinct in the time domain (separable events; Beerenwinkel et al., 2007a,b), datasets with identical combinations of events (identical columns) need to be pre-processed in order to collapse identical columns (events) in to one column (event).

#### RESIC

The graphs inferred by RESIC do not report nodes corresponding to events whose mutation frequency is greater than 80% (see Figure 1D and absence of the events 1, 2, and 3). RESIC allows to set two parameters, (i) the pairfreq (pf) that is the minimum co-occurrence frequency of mutations to consider, and (ii) res, the minimum marginal mutation frequency to consider. To improve the outputs from the binary triangular matrices those parameters need to be adjusted. Figure S9 demonstrates their effect on the number of predicted edges.

#### TO-DAG

Genes that are altered in all samples lead to nodes with outgoing edges only (i.e., null in-degree nodes) indicating independent events. In case all but one column (here the last) are entirely filled by 1s, the graph shows 19 independent events whose outgoing edges point to the twentieth event (see Figure 1E). In Figure S7 we observe how the distribution of the edge weights changes correspondingly to the step-wise increment of mutation frequency.

Assortativity and clustering coefficient as function of mutation frequency have been explored for the graphs inferred from inputs matrices of Figure 1A and then compared with the one on a random binary matrix of uncorrelated events of same size (20 samples × 20 mutations). Figure S8 shows the results and illustrates the behavior of these topological indices together with snapshots of the structure of the inferred networks taken at intermediate mutation frequencies. Figures S8A,C show results corresponding to mutation frequency range from 0.1 to 0.975 with an incremental step of 0.025. For each of the 36 mutation frequencies, a subset of 20 random binary matrices has been considered. The width of the error bars indicates the value of the standard deviations of the mean value of assortativity and transitivity, respectively. Figures S8B,D show the behavior of the indices calculated on the 20 matrices obtained from the lower triangular matrix by progressive filling of each row by 1s.

In Figures S8A,B we observe that the absolute value of assortativity ranges from 0 to 0.5 and decreases as the density of the connections decreases, i.e., as the number of independent nodes increases. By definition, assortativity is not quantifiable in the limit case in which *N*–1 nodes are independent (where *N* is the total number of nodes). Negative values of assortativity indicate the tendency of the nodes to preferentially connect with nodes of significant different degree.

Figure S8C on 20 by 20 input matrix confirms the results we showed in Figure S5A for the case of a 100 by 60 input matrix, and shows a low clustering coefficient at the extreme values of mutation frequency 0.1 and 0.975. For low average mutation frequency, as well as for very high average mutation frequency a small number of edges is inferred, as in the both case the method cannot determine the causal interdependencies between rare events or on high frequency and almost equi-probable events. Finally, Figure S8D shows the decrement of the transitivity also in the case of a step-wise increment of mutation frequency of the lower triangular matrix.

#### Disjoint datasets

**Figure 2:**
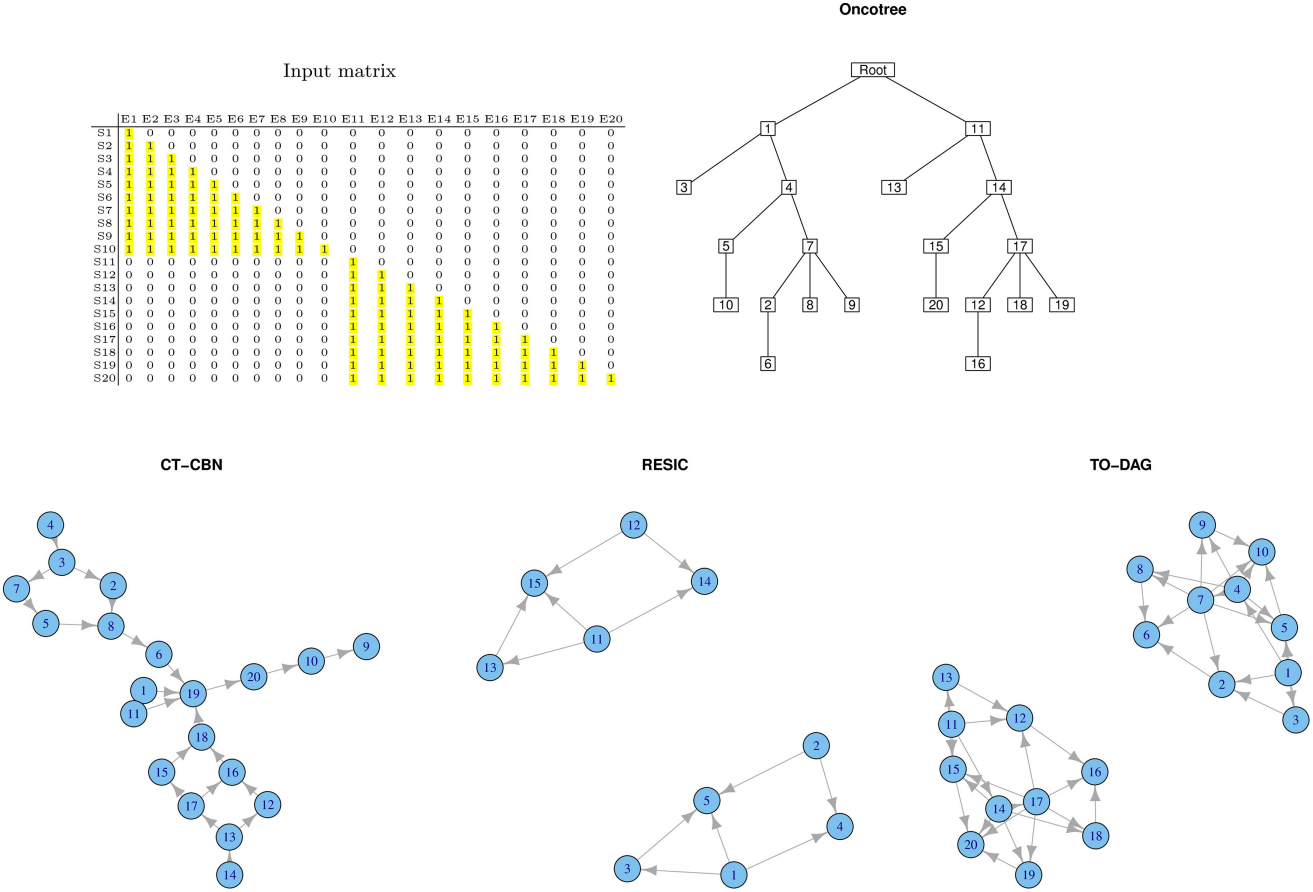
**Output graphs inferred from disjoint sample sets, i.e., set of samples that do not share any mutational event**.

Often real human tumor datasets include tumor sub-types or sub-classes (e.g., phenotypes, grades, stages) that might barely share any common genomic event. To mimic such a situation, we created a disjoint sample set (see Figure 2). Samples from S1 to S10 harbor genomic events only from E1 to E10 where samples from S11 to S20 only in events from E11 to E20. For each group of samples we chose a lower triangular matrix to facilitate the interpretation of the corresponding tree/network. The null intersection of the sets of events is reflected in the oncotree structure (two independent branches depart from the root), as well as in the RESIC and TO-DAG graphs that inferred disjoint sub-networks each one including the set of events defined in the disjoint sets of samples. CT-CBN fails to correctly recognizing such structure; E1 and E11, that are independent by the current experimental design, are both pointing to E19 that is a “collector node” for three sets of events: (2, 3, 4, 5, 7, 8), (20, 10, 9), and (12, 13, 14, 15, 16 17, 18). Moreover, events 15, 16, 17, and 18 (and also the set of events 12, 13, 16, 17) are connected in a loop so that it is not possible to establish a temporal order for these events.

#### Common genetic events in separate phenotypes

**Figure 3:**
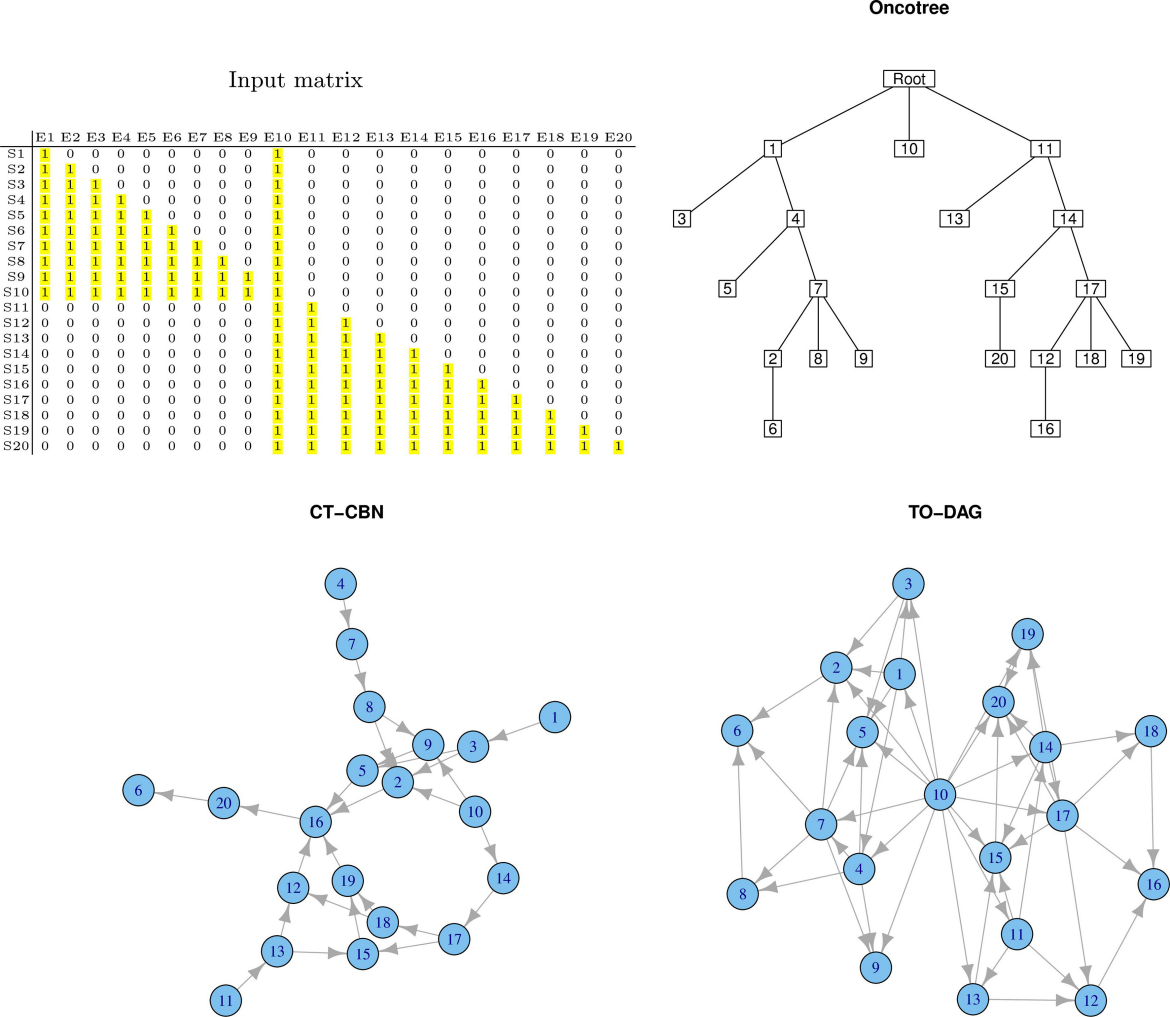
**Output graphs obtained from intersecting sample sets, i.e., set of samples that share at least one mutation event**.

If an event, say for example E10, is present in both samples sets, the corresponding node in the graph structure is expected to be an independent node connected to nodes relevant to both sample sets. Figure 3 exemplifies this experiment. TO-DAG identifies E10 as a hub node without incoming edges and connecting the two sub-networks related to events occurring in the two corresponding samples sets. The oncotree approach returns a structure where E10 is represented as an independent leaf directly originating from the root.

RESIC tested on a similar case study, where in the input matrix the E10 has been defined positive only in some samples (events positively defined in all the samples are removed from the graph as RESIC considered them as wild-type) does not include the event and returns two disjoint sub-networks.

#### Generation of *in silico* benchmarks and evaluation of performances

Next we turned to a more general assessment of the performance of TO-DAG and the other three selected methods in terms of accuracy of prediction on synthetic graphs with more complex topological structure. We utilized a procedure for *in silico* benchmark generation and performance profiling of graph inference based on the generation of gold-standard accessible pointed graph (APG) directed acyclic graphs and synthetic data (Johnsonbaugh and Kalin, 1991). An APG is a directed graph with a distinguished vertex (the “root”) such that every other vertex is reachable from it. That is, for every vertex in the graph, there is at least one path in the directed graph from the root to that vertex. We chose an APG as gold-standard topology, as it is a good compromise between the complexity of a realistic network of genetic events and the mathematical and computational complexity of the set of equations and constraints needed to generate corresponding binary matrix input to TO-DAG. Keeping this system of equations and constraints as simple as possible allows for a better control of the generation of indirect “relationship” between events and their strengths. It is very hard not to introduce indirect effects between nodes using equation-based modeling to describe network interactions. This problem is often overcome by introducing noise in the equation of a complex set of constraints on the mathematical relations among the nodes. If the topology of the gold-standard is very complex, the definition of this effect and the control of their impact on the graph inference performance become complex as well.

The procedure for the generation of a gold-standard APG is as follows.

1. Given the number of nodes n and of edges m, generate an unweighted random APG with n nodes and m edges. We used the C + + software package developed by Johnsonbaugh and Kalin (1991) to generate such sort of graph.
2. For each path starting from the root, assign a value to the joint probability of the events represented by the nodes in the path, i.e., for each path ***P***_***k***_, connecting ***n***_***k***_ nodes {_***v***_***i***_}***k***_ where ***i***=***1, 2, …, n***_***k***_ and ***k*** is the number of paths in the graph, define an arbitrary value of ***Prob***(**∩**_***v***_***i***_)***k***_
3. For each pair of nodes connected by an edge, define the constraints on the conditional probabilities of each node given its direct predecessor to model the directions defined in the topology.
4. Solve the sets of constrained equations to calculate the probabilities of the single events.
5. Generate the binary co-occurrence table with events/nodes on the column and samples on the rows, where the mutation frequency of each event is defined by the probability of the event calculated in step 4.

In Supplementary Material we illustrate this procedure on a small graph of 8 nodes. The performances of TO-DAG, CT-CBN and RESIC on synthetic APG gold-standards of increasing size and complexity have been evaluated in terms of Area Under the Receiver Operating Characteristic (AUROC). See Table 2. TO-DAG outperforms RESIC performances on networks of large size.

**Table 2:** **AUROC for the inference of TO-DAG, RESIC, and CT-CBN on synthetic gold-standards of different size**.

**# Nodes**	**# Edges**	**TO-DAG**	**RESIC**	**CT-CBN**
10	30	0.83	0.70	0.81
100	300	0.71	0.63	NA
1000	3000	0.85	0.69	NA

#### Experiments on genetic mutations from human tumor datasets

We selected two tumor types with high worldwide incidence, prostate cancer and melanoma, for which somatic genetic events datasets are available from whole genome and/or whole exome sequencing experiments of human tissues (Barbieri et al., 2012; Baca et al., 2013). Whenever the size of the input and/or the computational charge is manageable we also present the results obtained with CT-CBN and Oncotree.

#### Prostate cancer

**Figure 4:**
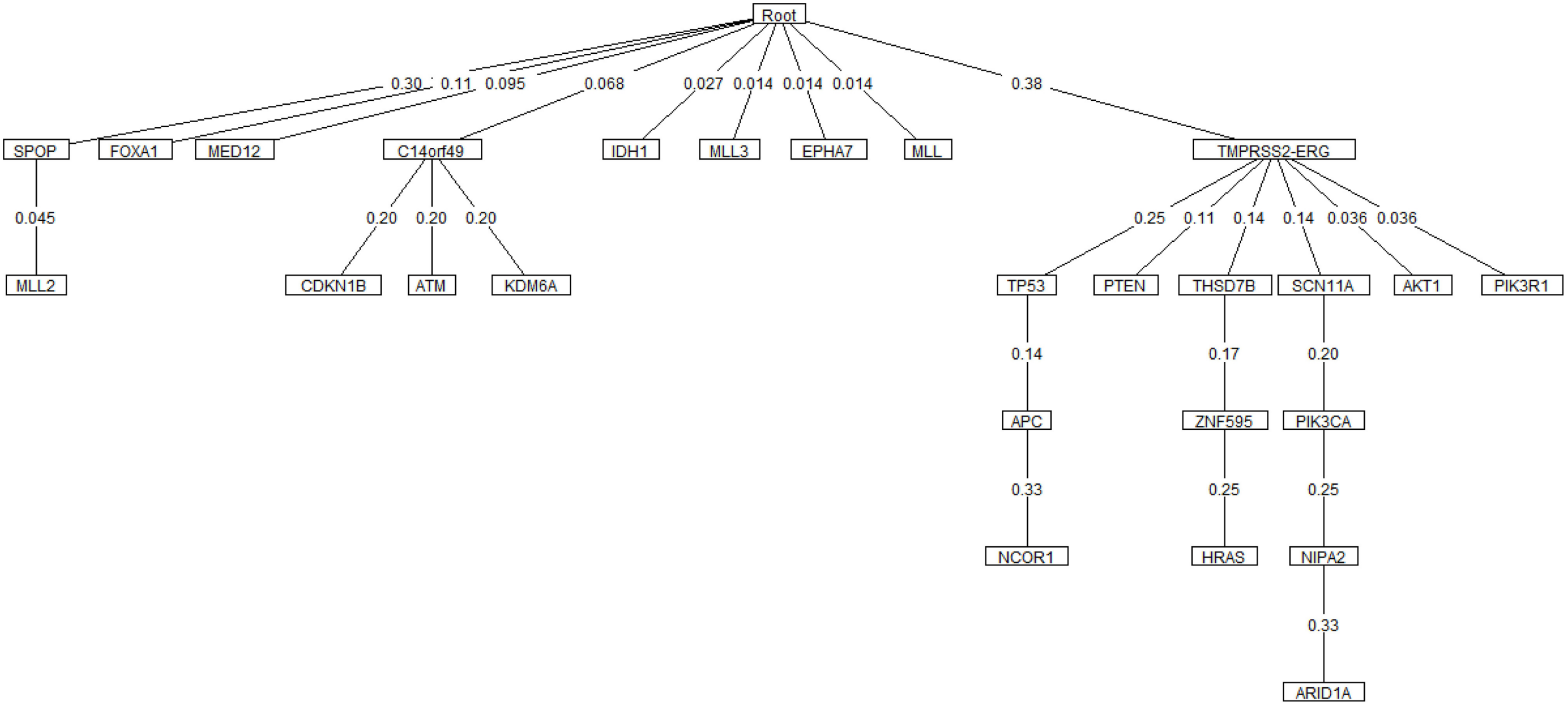
**Oncotree inferred from a prostate adenocarcinomas dataset (Barbieri et al., 2012)**.

**Figure 5:**
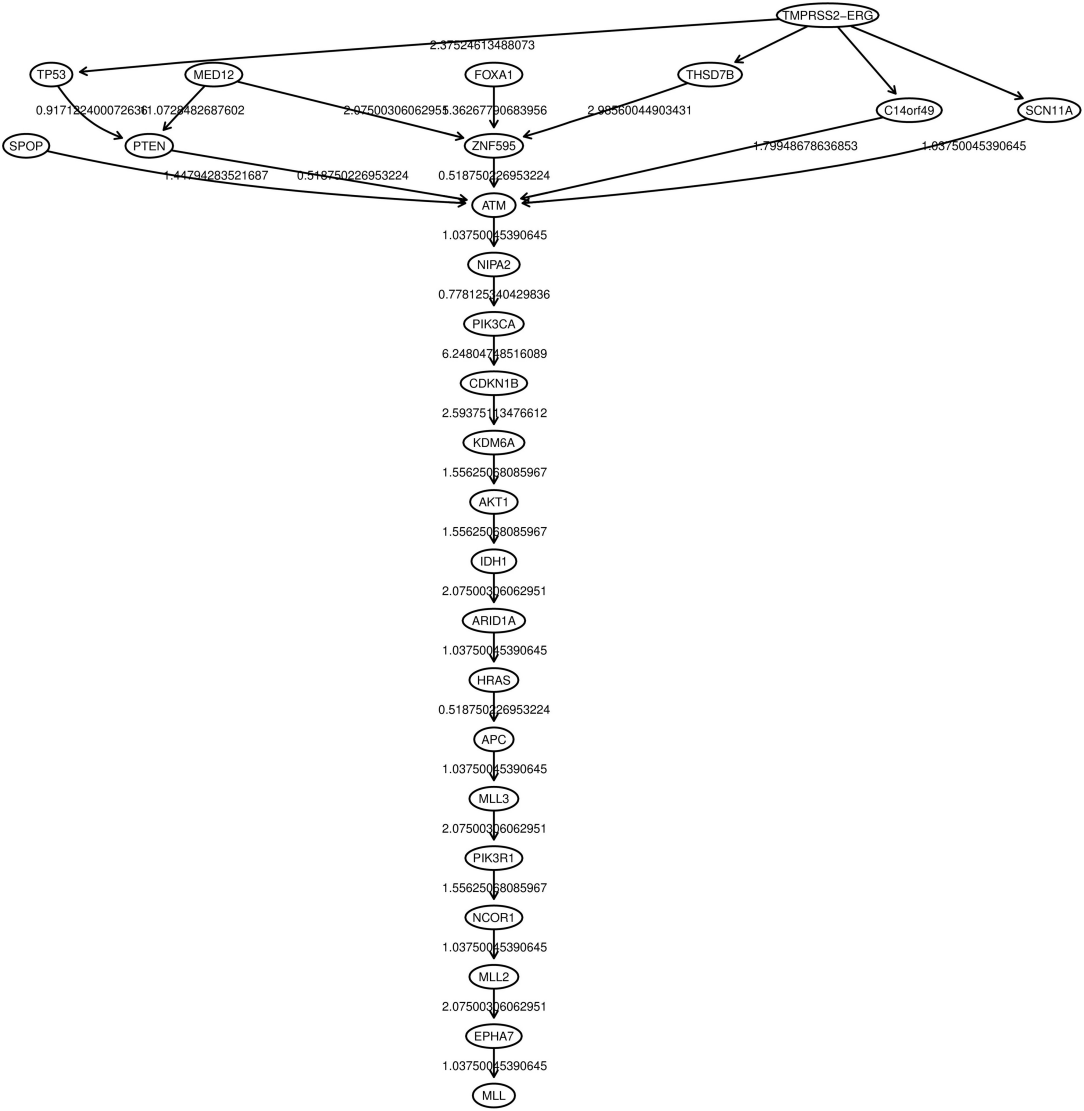
**CT-CBN graph inferred from a prostate adenocarcinomas dataset (Barbieri et al., 2012)**.

**Figure 6:**
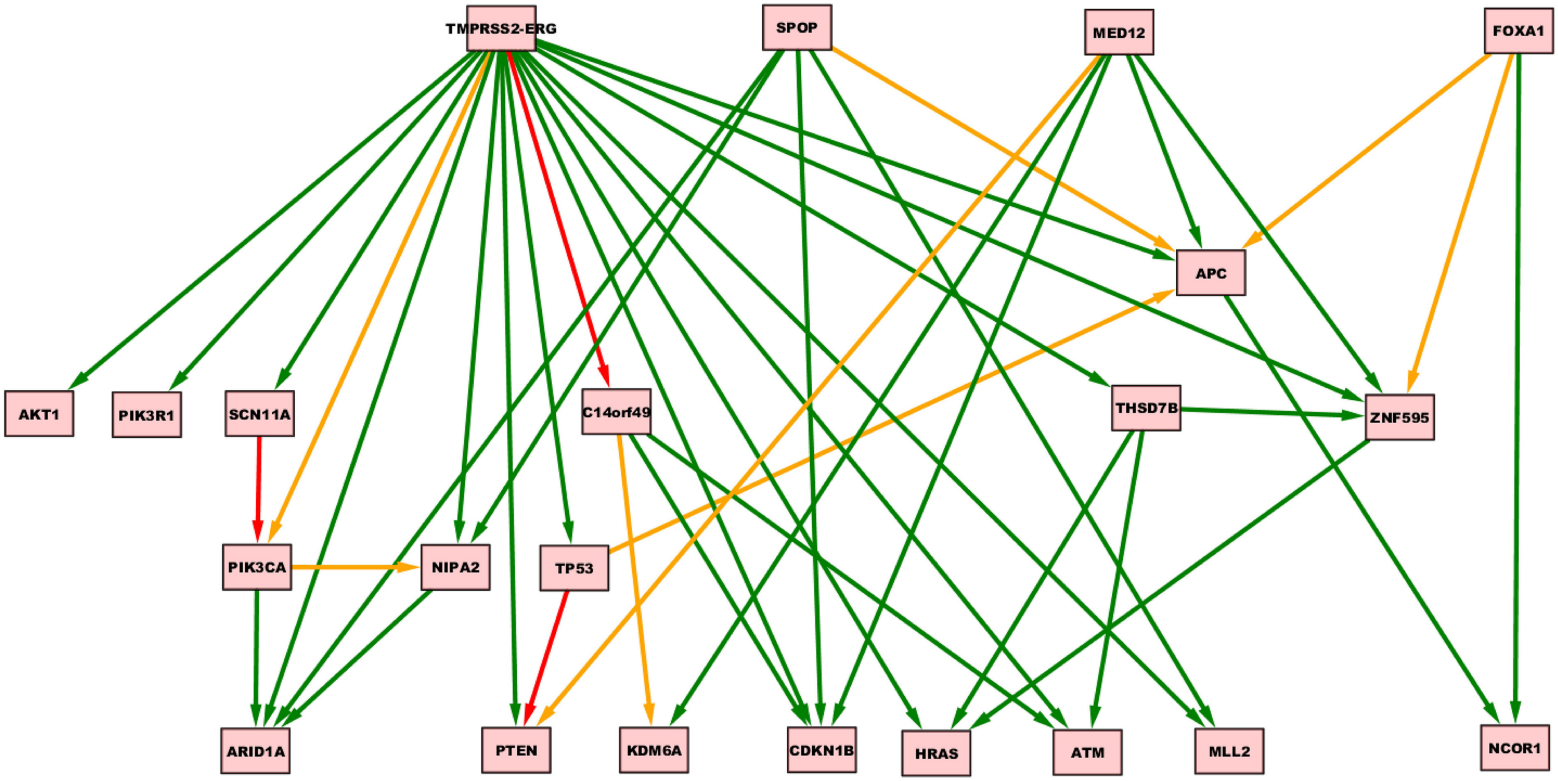
**TO-DAG model inferred from a prostate adenocarcinomas dataset (Barbieri et al., 2012)**. Green, orange, and red indicate fast (△*t* < 5), medium-speed (5 ≤ △*t* < 20) and slow (△*t*≥20) transitions. Waiting times are measured in arbitrary units (a. u.).

Oncotree, CT-CBN, TO-DAG performances have been tested on genetic data from a total of 74 patients. First, we considered a restricted set of 25 point mutations from the Barbieri study (Barbieri et al., 2012) plus the recurrent fusion event involving the *ERG* oncogene (Perner et al., 2007), an early event in prostate carcinogenesis enriched in young patients (Schaefer et al., 2013; Weischenfeldt et al., 2013). Figures 4–6 show the tree/graph structures inferred by Oncotree, CT-CBN and TO-DAG, respectively. As expected, all the approaches predicted that *ERG* fusion event is independent from any other event, but predicted different sets of *ERG* dependent events. For instance, CT-CBN output reports linear chains for events for which the algorithm did not infer a temporal order; RESIC predicts no inter-dependencies among the point mutations; where TO-DAG output resembles the expected order of selected events such as the dependency of *PTEN* from *TP53* consistent with the *PTEN* mutation being a late event during progression (Barbieri et al., 2012; Prandi et al., 2014). TO-DAG also inferred the relative velocity of the transitions distinguishing among slow, medium-speed, and fast transitions as per the waiting times (red, orange, and green edges, respectively; Figure 6).

**Figure 7:**
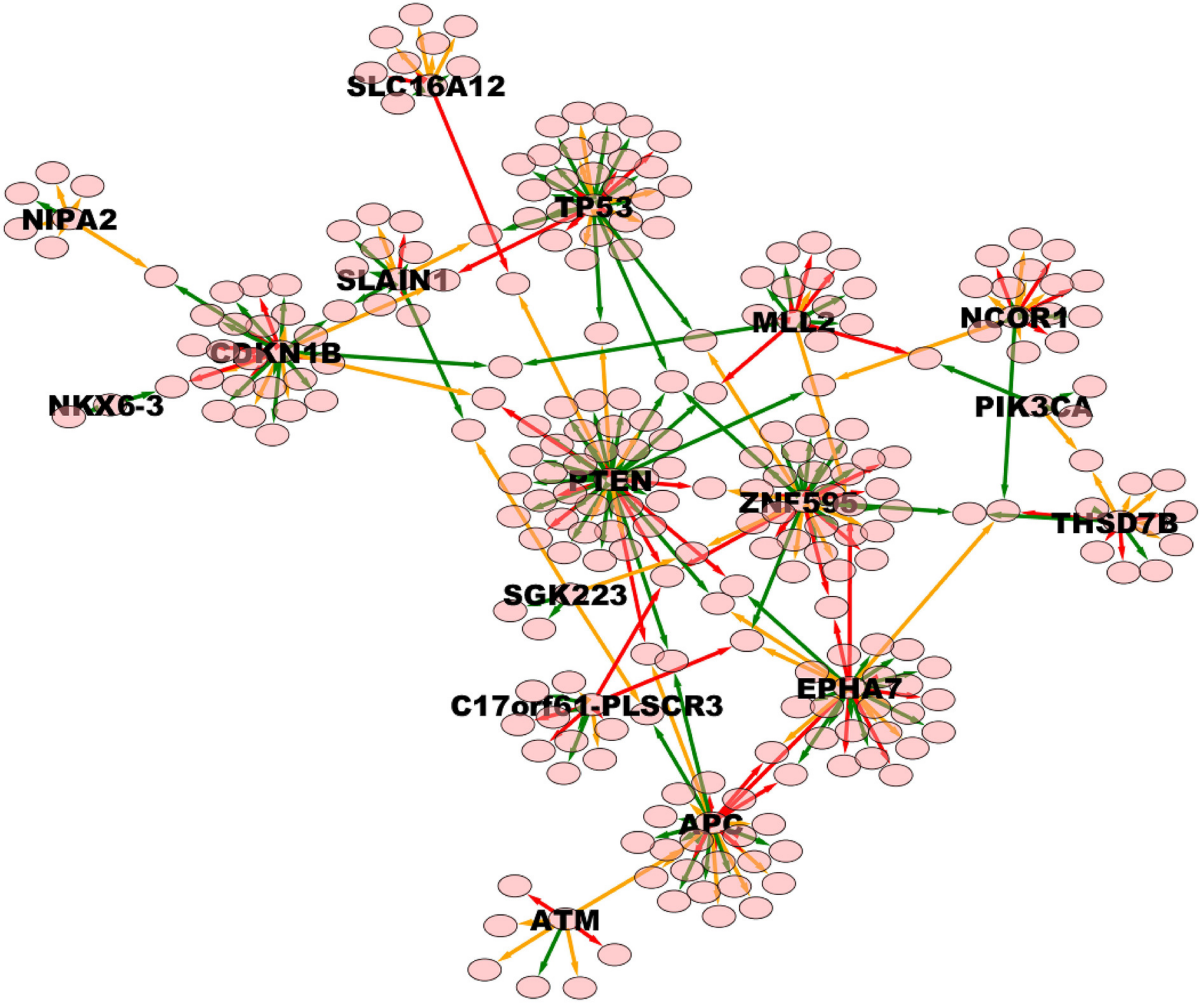
**High weight (equal or above 0.8) TO-DAG model from the prostate adenocarcinomas dataset (Barbieri et al., 2012)**. Green, orange, and red edges indicate fast, medium-speed, and slow transitions. Waiting times are measured in arbitrary units (a. u.). We refer the reader to the GraphML file graph_figure_7.graphml in Supplementary Data to visualize this network and to zoom in its details.

Next, we considered a larger dataset including a variety of genomic aberrations, namely copy number gains and losses, rearrangements, and point mutations (Baca et al., 2013). This set was analyzed with TO-DAG and with Oncotree. Given the complexity of the resulting networks, the Oncotree ones are presented in Supplementary Material. TO-DAG results are summarized in Table 3 that lists the number of samples, genes and predicted edges and in Figure 7 that illustrates a sub-network with edges weight greater than 0.8 and collapsed aberration types on a node/gene basis. Full information is retained in the GraphML format files in the Supplementary Data with nodes labels containing aberration suffixes (PM, BP, LOSS, GAINS). To gain an overall picture of the transitions, we calculated the percentage of slow, medium, and fast transitions in each network for any combination of source and target nodes labeled as PM, Point Mutation; L, Loss; G, Gain; and BP, Break-point structural rearrangements, as in Tables 4, 5. Of interest the small percentage of inferred slow transitions are between rearrangements (BP-BP) compatible with subsequent coordinated event sets that we had previously named chromoplexy (Baca et al., 2013) as a mechanism of punctuated evolution.

**Table 3:** **Size of the input dataset provided in Baca et al. (2013)**.

	**# Samples**	**# Genes**	**# Edges**
Gains	40	941	8338
Losses	48	948	7391
Point Mutions	48	961	6922
BP Rearrangments	54	3331	318.263

**Table 4:** **Percentage of slow, medium, and fast transitions in the network of Figure 6; PM, Point mutations; L, Losses; G, Gains; BP, Break-Point rearrangements**.

	**Total nr. of Edges**	**% of Fast**	**% of Medium**	**% of Slow**
PM↔PM	1567	53	44	3
PM → L	1171	54	43	3
L → PM				
PM → G	262	54	41	5
G → PM				
L↔L	135	58	39	3
L → G	79	2	49	49
G → L				
BP → PM	158	44	52	4
PM → BP				
BP → L	0	0	0	0
L → BP				
BP → G	0	0	0	0
G → BP				

**Table 5:** **Percentage of slow, medium, and fast transitions in the network of Figure 7**.

	**Total nr. of Edges**	**% of Fast**	**% of Medium**	**% of Slow**
BP↔BP	318.263	1	1	98
G↔G	8338	84	15	1
L↔L	7391	64	31	5
PM↔PM	6922	61	35	4

*PM, Point mutations; L, Losses, G, Gains; BP, Break-Point rearrangements*.

**Figure 8:**
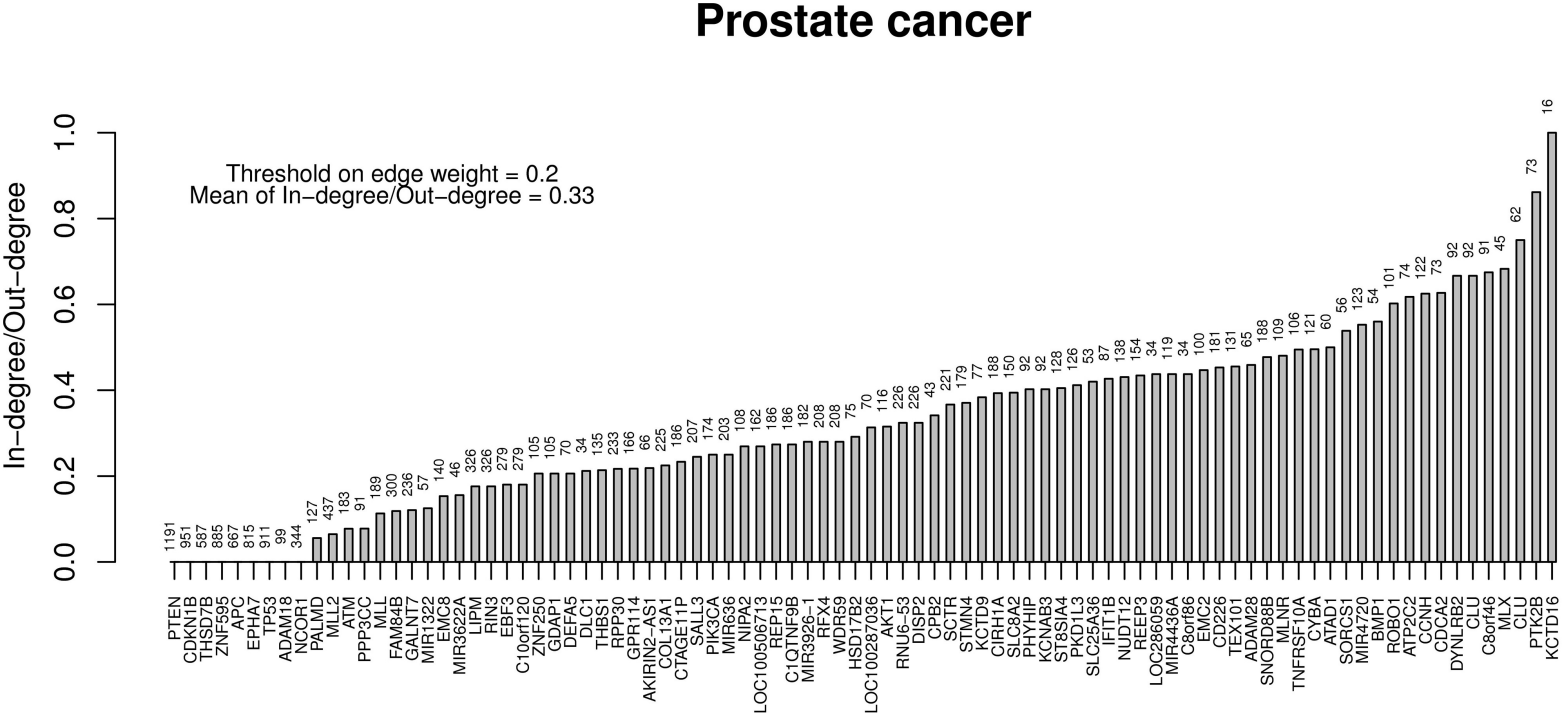
**Ratios between in- and out-degrees of prostate cancer genes (i.e., network nodes from Figure 7)**. Genes with out-degrees equal to zero are not included. Values on top of each bar indicate the total node degree. For a comparison with the melanoma analysis, see in the text.

Finally, Table 6 reports the in- and out-degree of the hub nodes of the network, defined as nodes with Kleinberg's centrality greater than 0.8 for different values of edge weight threshold. Interestingly, the analysis highlights key tumor suppressor genes, both gatekeepers and caretakers (Kinzler and Vogelstein, 1997) such as *TP53, PTEN*, and *CDKN1B*, as having the smallest in- over out-degree ratios or, in other words, as genes that when mutated behave like mutation firework initiators (Figure 8). In the Supplementary Material, we provide both in GraphML and tab delimited three subgraphs of the whole network from Figure 7 (available graph_figure_7.graphml, Supplementary Data) only including “slow” transition among nodes, “medium-speed” transition among nodes, or “fast” transition among nodes.

**Table 6:** **In- and out- degrees of the hub nodes (i.e., nodes with Kleinberg's centrality greater than 0.8) of the network of genetic events in prostate cancer showed in Figure 7 for different values of the threshold on the edge weight**.

**Cutoff**	***CDKN1B***		***EPHA7***		***PTEN***		***APC***		***TP53***		***MLL2***		***ATM***		***NCOR1***		***ZNF595***	
	**In**	**Out**	**In**	**Out**	**In**	**Out**	**In**	**Out**	**In**	**Out**	**In**	**Out**	**In**	**Out**	**In**	**Out**	**In**	**Out**
>> 0.8	0	27	0	29	0	43	0	25	0	30	0	15	0	7	0	15	0	34
0.7	0	872	0	756	16	1076	0	658	0	830	0	367	0	179	0	265	11	822
0.6	0	900	0	781	16	1107	0	662	0	858	0	381	0	181	0	282	11	830
0.5	0	901	7	794	16	1114	0	670	0	875	0	394	0	182	0	322	11	830
< < 0.2	0	951	7	815	16	1191	0	677	0	911	0	433	2	187	0	344	11	885

*For non-hub nodes, the average ration between in- and out-degree is 0.75*.

**Figure 9:**
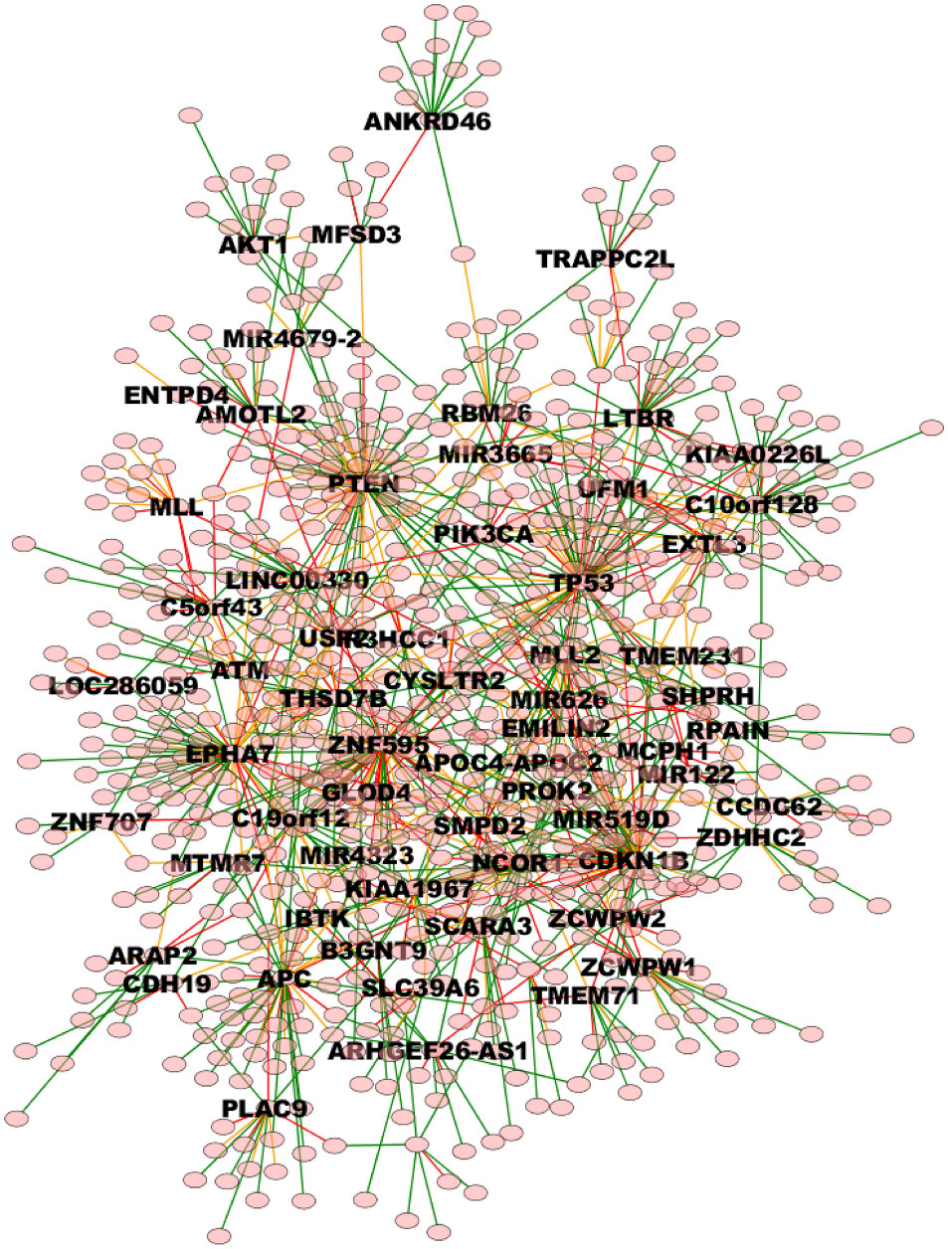
**TO-DAG model graph inferred from a dataset including a variety of genomic aberrations, namely copy number gains and losses, rearrangements, and point mutations (Baca et al., 2013)**. This network shows edges whose weight is greater or equal than 0.4. Only names of the nodes with hub score above the average are reported. As a consequence, it presents more edges than the network in Figure 7. Green, orange, and red edges indicate fast, medium-speed, and slow transitions. The majority of medium-low scoring hubs are connected through more than one node. The probability of this interconnection is slightly lower than the average. Waiting times are measured in arbitrary units (a. u.). We refer the reader to the GraphML file graph_figure_8.graphml in the Supplementary Data to visualize this network and to zoom in its details.

The whole TO-DAG network (including all edges with probability greater than the mean value 0.4) is shown in Figure 9 (graph_figure_9.graphml, Supplementary Data).

#### Melanoma

The genomic landscape of melanoma is characterized by a large amount of point mutations. Recent work from Berger et al. (2012) on 25 metastatic melanomas identified key genes that are significantly mutated including *BRAF* and *NRAS*. TO-DAG inferred a graph with 5139 nodes and 1129,295 edges from all 4917 melanomas protein coding point mutations. To assess the validity of the these measures and, indirectly, to quantify the deviation of the TO-DAG inference from the randomness, we focused on the set of key genes and compared their centrality measures (degree and node strength, Table 7) with those obtained in TO-DAG graphs inferred from random input matrices having the same size and the same density (i.e., the same number of positive entries) of the real input matrix. In Table 8 the mean values of minimum, maximum, mean, median, first and 3rd quartiles of in- and out-degree distributions obtained from the 100 random experiments are shown. We found that out- degree for the genes of interest are between the mean value of the 3rd quartile and the maximum except for *NRAS, GOLGA6L6, OR2T33*, and *MST1*, whereas the in- degree and node strength are between the mean minimum and the first quartile value, except for *NRAS* and *OR2T33*. Next, we inferred putative causality networks from a reduced data set including only cancer genes, and compared the in- and out-degree of *BRAF* and *NRAS* with the distribution of in- and out-degree of these genes obtained from 100 TO-DAG inferences from 100 binary matrixes where the mutation frequency of each single gene is kept constant and equal to the real data. Results are shown in Table 9. The degrees in real cases deviated from the mean values of the random case, that are D_*BRAF*_°^*ut*^ = (178.94 ± 9.21) and D^*in*^_*NRAS*_ = (15.01 ± 4.07).

**Table 7:** **Mutation frequency, degree, and node strength of significantly mutated genes (Berger et al., 2012) as in TO-DAG inferred graph on melanoma case study**.

**Gene**	**Mutation frequency**	**Out-degree**	**In-degree**	**Out-Strength**	**In-Strength**	**Out-degree/Out-Strength**	**In-degree/In-Strength**
*BRAF*	0.391	2830	6	2002.21	0.3704	0.7075	0.0617
*NRAS*	0.043	0	514	0	514	NA	1.0000
*PREX2*	0.478	3639	1	2762.70	0.0744	0.7592	0.0744
*GOLGA6L6*	0.174	952	67	624.05	6.25	0.6555	0.0933
*VCX3B*	0.174	1022	70	693.72	6.375	0.6788	0.0911
*POTEH*	0.217	1589	42	1049.83	3.56	0.6607	0.0848
*OR2T33*	0.087	278	159	278	44.25	1.0000	0.2783
*C1orf127*	0.217	1969	39	1387.33	3	0.7046	0.0769
*PRG4*	0.345	2628	7	1861.11	0.4219	0.7082	0.0603
*MST1*	0.217	1332	44	857.75	3.56	0.6440	0.0809

**Table 8:** **Average quartiles of the in-degree and out-degree distribution the distributions have been obtained from 100 TO-DAG graphs inferred from 100 random binary matrices of the same size and density of the real data matrix in Berger et al. (2012)**.

**Quartiles**	**In-degree**	**SD of in-degree**	**Out-degree**	**SD of out-degree**
Min.	0	0	0	0
1st	144.87	2.03	0	0
Median	156.54	2.1	0	0
Mean	151.08	0.13	151.08	0.13
3rd	166.5	1.75	305.22	3.23
Max.	184.83	5.58	4120.2	28.76

**Table 9:** **In- and out-degree of BRAF and NRAS genes in TO-DAG network inferred from Cancer Genes list. (A) Summary of the distributions of in-degree (B) and out-degree (C) obtained from 100 TO-DAG networks inferred from 100 random binary matrices, where the mutation frequency of each single gene is kept equal to the real one**.

	**Out-degree**					**In-degree**
**(A)**						
*BRAF*	213					0
*NRAS*	0					60
	**Min**.	**1**° **Qu**	**Median**	**Mean**	**3**° **Qu**	**Max**
**(B)**						
*BRAF*	0	0	0	0	0	0
*NRAS*	6	12	15.5	15.91	18	26
**(C)**						
*BRAF*	160.0	172.0	179.0	178.9	187.2	204
*NRAS*	0	0	0	0	0	0

All genes showed out-degrees larger than in-degrees in line with their significant mutation frequency and the mathematical definition of TO-DAG weights. The only exception was *NRAS* that exhibited null out-degree and in-degree equal to 514. When comparing the *NRAS* induced sub-graphs of order 1 with the *NRAS* pathway from Pathways Commons PPI database (http://www.pathwaycommons.org/about/; 1030 nodes and 388,000 edges) we found 20 direct interactions of *NRAS* present in both graphs (Table 10) including a link from *BRAF* to *NRAS*.

**Table 10:** **Edges in the intersection of TO-DAG ***NRAS*** sub-graph of order 1 (514 edges and 513 nodes) and ***NRAS*** pathway as reported in Pathways Commons (1030 nodes and 388,000 edges)**.

**Source**	**Target**
*MLLT4*	*NRAS*
*SPTA*	*NRAS*
*KLB*	*NRAS*
*RASA2*	*NRAS*
*PIK3C2G*	*NRAS*
*CSF2RB*	*NRAS*
*BRAF*	*NRAS*
*ANGPT1*	*NRAS*
*KIF20B*	*NRAS*
*FLNB*	*NRAS*
*EPS8*	*NRAS*
*DYNC1H1*	*NRAS*
*DST*	*NRAS*
*DIDO1*	*NRAS*
*CPNE3*	*NRAS*
*ANKRD11*	*NRAS*
*NF1*	*NRAS*
*MYOF*	*NRAS*
*EXOC4*	*NRAS*
*CKAP5*	*NRAS*

*477 nodes/genes present in the whole network of the melanoma case study (Berger et al., 2012) are not reported in the NRAS pathway*.

**Figure 10:**
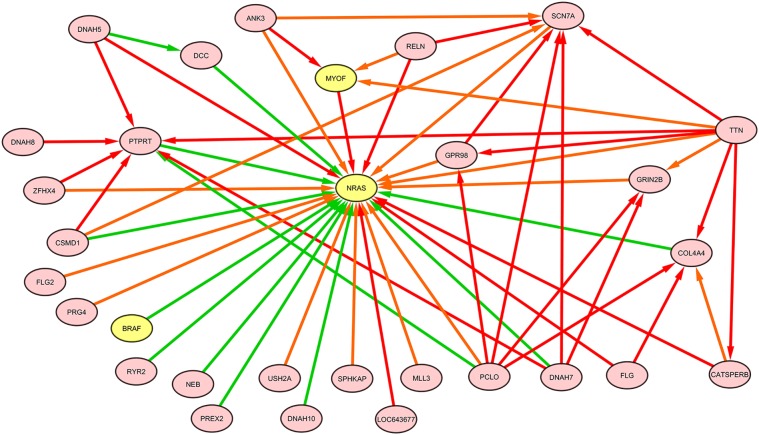
**NRAS induced sub-graph of order 2 extracted from the TO-DAG model inferred from a metastatic melanoma dataset including somatic base pair mutations in protein coding regions (Berger et al., 2012)**. Of the *NRAS* induced sub-graph of order 2 containing 30 nodes and 378 edges, only edges with weight above the mean value (0.15) in the subgraph are shown in this figure.

The *NRAS* induced sub-graphs of order 2 (30 nodes and 378 edges) extracted from the whole melanoma graph is provided as a GraphML file (NRAS_order2_subG.graphml, Supplementary Data); Figure 10 shows the sub-graph restricted to edges with weight greater than the mean value (equal to 0.15).

Where these large network results are exploratory in nature and should be followed by independent validation, they provide the first time directed networks from large human sample mutational datasets. These analyses can highlight nodes and bottlenecks during tumor evolution that can inform key elements in cancer progression.

## Conclusions

We presented TO-DAG, a new tool suitable to model non-memoryless process of mutations accumulation, to handle very large datasets and to estimate the waiting time of transitions from the occurrence of a mutation to the occurrence of the subsequent one. TO-DAG competes with the current DAG and tree models, and in particular with the more complex Bayesian models, in the accuracy of estimating the order and the waiting time of mutation events. The theoretical model of the majority of graphs is based on pairwise dependencies between genetic events. Furthermore, the majority of timed graph models assume that mutations are random events and that the process of their accumulation is a memoryless stochastic process. Due to these assumptions, current graph models are a simplistic generalization of the tree models and allow only the specification of stochastic process having the Markov property. Such property holds when the conditional probability distribution of future states of the process (conditional on both past and present state) depends only upon the present state, not on the sequence of events that preceded it.

TO-DAG discards the Markov property assumption and is not limited to compute “pairwise” dependencies between genetic events. As a consequence, the new probabilistic theory of TO-DAG allows for the inference of pathways of causal dependencies among genetic alterations more closely reflecting the dynamics of the mutation accumulation process during cancer progression. Such probabilities can be estimated directly from the data, and the waiting times of the mutation events are estimated a posteriori as stochastic function of their conditional probability. In the TO-DAG inferential framework the probability of occurrence of a mutation is not a function of the time. The reason of such a probability model is the impossibility to measure the exact time at which a mutation occur. The estimation of the waiting time of mutation is carried out once the causal dependency topology of the graph has been determined. Finally, since no a priori assumptions about the order and the timing of mutation accumulation process is needed and no parameters other than the confidence level γ are needed as input of the inferential procedure, the TO-DAG faithfully represent the topological structures embedded in the input data.

A comprehensive comparison of the results obtained with TO-DAG with approaches from the three main categories of mutation patterns inference has been presented and discussed in terms of five main aspects: (1) the computational complexity and the subsequent ability to successfully deliver an output from huge amount of data (compatible with the current state of the art in cancer genomics), (2) the outputs from random data to estimate the number of putative false positive as function of the number of genetic events, number of samples, and mutation frequency; (3) the outputs from synthetic data generated by a step-wise increase of the mutation frequency to assess how and the extent to which each inference model reflects the input data structure in a graph; (4) the outputs from data in which sample set do not share (and do share) positive occurrences of genetic events to assess the ability of the methods to infer disjoint (joint) graphical models corresponding to disjoint (joint) sample sets, and (5) the assumption and approximation that each method adopts in its theoretical framework (e.g., memoryless property, removal or collapse in unique events of aberrations positively in all samples).

Oncotree and TO-DAG exhibited similar good performances across multiple comparisons (1–4), however thanks to the graph based approach TO-DAG outperforms Oncotree whenever a slightly complex topology is involved and requires nodes with more than one parent and/or when parents are connected. The former especially is crucial in cancer genomics as independent paths can lead to the same crucial event. TO-DAG output exactly reflects complex topological structure embedded in the synthetic data. Indeed all the expected interactions are correctly predicted. Oncotree, RESIC and TO-DAG infer disjoint subnetworks corresponding to disjoint sample sets, whereas CT-CBN revealed a strong limitation on this case study. Moreover, CT-CBN, given the complexity of mathematical structure formalizing a Bayesian inference model, is limited to few tens of input events and samples and to an average/low sparseness of the data. RESIC is very well performing with regard to aspect 2, as it has a very low rate of false positive predictions with respect to TO-DAG and CT-CBN.

Furthermore, TO-DAG can process huge datasets and implements a new theoretical model in which the accumulation of genetic events during cancer development is a probabilistic timed non-memoryless process. In contrast with the majority of DAG-based methods (especially the Bayesian ones), TO-DAG is not parametric and does not require any a priori knowledge on the causal dependencies among genetic events; TO-DAG inferred topologies are deduced only from the conditional probabilities estimated from the data.

Finally, TO-DAG represents a solid alternative to sequence based evolutionary approaches that ultimately utilize lesion clonal state or allele frequency information to construct evolution charts (Carter et al., 2012; Prandi et al., 2014). TO-DAG is readily applicable to mutation or aberration data irrespectively from the experimental platform used to generate the data.

Altogether, the formal comparison of available approaches and the introduction of new methods able to deal with state-of-the-art genomic datasets improves our ability to make the best use of the current genetic information eventually resulting in the identification of suitable drug targets relevant to tumor initiation and progression.

## Author contributions

PL and FD designed the TO-DAG method. PL implemented the TO-DAG algorithm. FD and NC contributed to the design of validation and performance testing methodologies. FD was responsible for the overall project. All authors equally contributed in writing and reviewing the manuscript.

### Conflict of interest statement

The authors declare that the research was conducted in the absence of any commercial or financial relationships that could be construed as a potential conflict of interest.

## Supplementary material

The Supplementary Material document includes details about the theoretical foundation of Oncotree, CT-CBN, and evolutive algorithm RESIC; the method for the *in silico* validation of TO-DAG, and supplementary figures and legends.

We also provide as Supplementary Data, the GraphML and tab delimited files of the TO-DAG inferred graphs from Figures 6, 7, 9 and for *NRAS* sub-graph (order 2), at the following link: http://demichelislab.unitn.it/TODAG
