# Supplementary material for: Defining order and timing of mutations during cancer progression: the TO-DAG probabilistic graphical model
Source: Front Genet. 2015 Oct 13;6:309. doi: 10.3389/fgene.2015.00309 (PMC4602157; doi:10.3389/fgene.2015.00309)
Supplement: Supplementary file 1 [file Presentation1.PDF]

SUPPLEMENTARY MATERIAL

Defining order and timing of mutations during cancer progression:

the TO-DAG probabilistic graphical model

Paola Lecca [1*], Nicola Casiraghi [1], Francesca Demichelis [1,2*]

[1] Centre for Integrative Biology, University of Trento, Laboratory of Computational Oncology, via Sommarive 9, 38123 Trento, Italy
Phone: +39 0461 285305, Fax: +39 0461 282742

[2] Institute for Computational Biomedicine, Department of Physiology and Biophysics, Weill Medical College of Cornell University, New York, NY 10021, USA

* Corresponding authors: Paola Lecca (paola.lecca@unitn.it), Francesca Demichelis (demichelis@science.unitn.it)

1 Methods to infer order and causal relationships among genetic alteration events

Here we report the mathematical foundations of the three methods we used to performances comparison against TO-DAG. The notation is as in the original manuscripts.

1.1 Oncogenetic trees

A tree is defined by $T = (V, E, r)$, where V is the set of nodes representing the genetic events, $E \subseteq V \times V$ is the set of directed edges, representing relationships between events, and $r \in V$ is the root of the tree, representing the starting point of the disease. A directed edge is represented by an ordered tuple (u, v), with starting point u and end point v. There is a directed path from r to every node and the root has no incoming edges. A quantitative oncotree can be obtained by adding weights to the edges. In a weighted (also said "labelled") oncotree $T_w = (V, E, r, w)$, w is a function $w : E \to \mathbf{R}$, such that $w(e) > 0$, $\forall w \in E$. By means of a labelled tree, one can characterize a probability of a path S in the following way:

$$p(S) = \prod_{e \in E'} w(e) \prod_{e=(u,v) \in E, u \in S, v \notin S} (1 - w(e)) \quad (1)$$

where $E' \subseteq E$ such that S contains all nodes reachable from the root $r \in S$ in the rooted tree (V, E', r). The weights $w(e)$ are the conditional probabilities of edges, which lead to the observed event. To specify these probabilities edge weights are defined for every combination of events based on the relative frequencies estimated from the co-occurrences data. Then, Edmond's branching algorithm is used to find a maximum or minimum optimum branching (Parson, et al., 2008; Gerstung, Eriksson, Lin, Vogelstein, & Beerenwinkel, 2011).

Here we report simple explicative examples of the application of formula (1). Consider the third oncotree in Figure S1B, and assume an observation $x = \{1, 9, 1, 1, 0\}$, that corresponds to the observation of the path of events E1, E3, and E4. The probability of this path is

$$p(r, \mathbf{x}) = 0.94 \times 0.67 \times 0.37 \times (1 - 0.2) \times (1 - 0.5) = 0.0932104$$

The first three terms represent the three observed events E1, E2, and E4, and the last two terms the non-occurrence of the events E2 and E5. As a second example assume an observation $x = \{1, 1, 0, 0, 1\}$, corresponding to the observation of path of events E1, E2, and E5. The probability of this path is zero, because event E5 can be observed only if event E3 has been observed.

These examples reveal that the oncogenetic tree allows for multiple pathways and can model disease progression in a more flexible way than the linear path models. However, in oncogenetic tree models, the possibility of occurrence only depends on the direct predecessor and only one parent is allowed. As noted by Hainke et al. (Hainke, Rahnenfuerer, & Fried, 2012) and Desper et. al (Desper R. , Jiang, Kallioniemi, Moch, Papadimitriou, & Schaffer, 1999) both the assumptions make the modelling very simple but are in fact questionable and might impede the ability to accurately capture the dominant factors of oncogenesis. There is a further subtle drawback of the oncotree model: the model cannot explain certain patterns of genetic events whether they have null probability. This limitation implies that the tree structure can represent disease progression only

for a subset of tumor/patients (i.e. the rows of the co-occurrence table).

Distance-based trees and oncogenetic tree mixture models have been developed to overcome these limitations. Distance-based oncotrees are tree structures representing the genetic events by leaf-nodes of leaves, which are nodes without children. The nodes between root and leaves are inner nodes indicating unknown events that cannot be observed. In this structure each combination of events has positive probability (i.e. $p(S) \geq 0 \; \forall \; S \subseteq V$) and thus it is informative of the relationships between every pair of events. Unlike the oncotree models, distance-based oncotrees do not provide an order of occurrence of the events, but as observed by Desper et al. (Desper R. , Jiang, Kallioniemi, Moch, Papadimitriou, & Schaeffer, 2000), since probabilities are multiplicative from root to leaf, the negative logarithm of edge probabilities can be used for a path edge. The sum of these values along the path from the root to a certain leaf measures the distance between start and event (Hainke, Rahnenfuerer, & Fried, 2012). The temporal order of the events can be deduced by comparing these distances, the smaller the distance the earlier the occurrence.

Oncogenetic tree mixture models have been proposed by Beerenwikel et al. few years ago (Beerenwinkel et al., 2005). The models represent different genetic mutational processes, which cause the disease progression by different oncotrees. To assure a non-null probability for every combination of events, the first tree component is modelled like a "star". In a star-like structure every node representing a genetic events directly emanates from the root and can therefore occur independently from all others. Hainke et al. (Hainke et al., 2012) point out that one can model the star component with equal probabilities (e.g. equal edges weights for every event) but also with non-equal probabilities.

Namely, tree mixture models include several tree components, each for one genetic mutation process, and each with a different weight. Weights are estimated exploiting optimization algorithms inspired to Expectation-Maximization methods (EM algorithms): for every sample of genetic events, the probability of belonging to a certain tree component is estimated in the E-step, whereas the single tree components are estimated in the M-step with the Edmond's branching algorithm. Although tree mixture models offer undoubted advantages in comparison to the oncogenetic trees, they are still tree-like modes, where there is one node per event and no cycles, the occurrence of a child event depends on the occurrence on its parents and no multiple parents are allowed.

1.2 Bayesian networks

Graphs are structures that allow for any network structures. Studies dating back to some years ago (Merlo, Pepper, Reid, & Maley, 2006; Sjoblom, et al., 2006; Vogelstein, et al., 1988; Desper R. , Jiang, Kallioniemi, Moch, Papadimitriou, & Schaffer, 1999) propose to adopt direct acyclic graph to model the occurrence of genetic mutation events responsible for cancer onset and progression. A directed acyclic graph (DAG) is a directed graph with no directed cycles. DAGs are formed by a collection of nodes and directed edges, each edge connecting one vertex to another, such that there is no way to start at some node A and follow a sequence of edges that eventually loops back to A again. Formally, Bayesian networks are directed acyclic graphs whose nodes represent random variables in the Bayesian sense: they may be observable quantities, latent variables, unknown parameters or hypotheses. Edges represent conditional dependencies; not connected nodes represent variables that are conditionally independent of each other. Each node is associated with a probability function that takes as input a particular set of values for the node's parent variables and gives the probability of the variable represented by the node. Beerenwinkel et al. (Beerenwinkel et al., 2007) introduced Conjunctive Bayesian networks (hereafter CBNs) to model

networks of mutations in carcinogenesis. CBNs are a class of Bayesian graphical models that describe the accumulation of events constrained to the order of their occurrence.

CBNs assume that the genetic changes are permanent and irreversible. Each individual (or tumor type) is completely defined by its genotype, i.e. by the subset of the events that have occurred. Inferential Bayesian approaches are used to learn the constraints on the orders in which these events have accumulated. A CBN is a probabilistic model of this process derived from a partial order on the set of events that encapsulates the dependencies between events (Beerenwinkel et al., 2007). In two papers, one in 2009 and the other in 2011 (Gerstung et al., 2011), Beerenwinkel, Gerstung and co-workers expose the theoretical foundations and the assumptions of the CBN models of oncogenesis. We will resume here their approach and we will use their notation.

Let $X = \{X_1, X_2, \ldots, X_N\} \in \{0,1\}^N$ be the vector of binary random variables, each indicating one of N fixed genetic events. In (Gerstung et al., 2009;Gerstung et al., 2011) the waiting time of mutations, denoted by the vector $T = \{T_1, T_2, \ldots, T_N\}$, is a random variable too, and it is required that all predecessor events of a mutation have already occurred before the mutation can occur. The time of diagnosis T_s is also introduced and is considered a random variable as well. Since the relation between T and T_s is generally unknown, the authors assume that T_s is independent of T. A set $pa(i)$ denoting the mutations that need to be present in order to set *i* is introduced. This set is a partially ordered set and defined as poset. A poset consists of a set together with a binary relation indicating that for certain pairs of elements in the set, one of the elements precedes the other. Such a relation is called "partial", because some elements in the set are not linked by a temporal order relationship. For instance it may be that neither element precedes the other in the poset. A poset together with the cover relation $j \to i$ for $i \in pa(i)$ compose the algebraic structure describing the network of genetic mutations and their relationships. The conditional density of X factorizes as follows

$$\text{Prob}_{\lambda,P}[X|T,T_s] = \text{Prob}_{\lambda,P}\left[\max_{i:X_i=1} T_i < T_s < \min_{j:X_j=0} T_j\right] \tag{2}$$

and the probability of X can be decomposed also in Bayesian factors as following

$$\text{Prob}[\mathbf{X}] = \int_0^\infty \int_0^\infty \text{Prob}[\mathbf{X}|T=t, T_s=t_s] \cdot f(t) f(t_s) dt dt_s \tag{3}$$

where $\text{Prob}[X=1|T=t, T=t_s] = \mathbf{I}(t \le t_s)$ and $\mathbf{I}$ is the indicator function (i.e. the function that is equal to 1 is $t \le t_s$ and 0, otherwise); $f(t)$ and $f(t_s)$ are the densities of the random variables t and t_s respectively.

The waiting times of mutations are assumed to be exponentially distributed:

$$T_i \sim \text{Exp}(\lambda_i) + \max_{j \in \text{pa}(i)} T_j$$

reflecting the underlying assumption that the accumulation of mutations is a Markov process. A stochastic process has the Markov property if the conditional probability distribution of future states of the process depends only upon the present state, not on the sequence of events that preceded it. Markov property is often referred to the memoryless property of a stochastic process, such as the possibility to make predictions for the future of the process based solely on its present state, and forgetting the past. We will comment later in this paper about the extent of validity of this assumption. The density of T_i conditioned to all predecessors $\{T_i\}_{j \in pa(i)}$ is

$$f_{T_i|\{T_i\}}(t_i|\{t_j\}) = \lambda_i(t_i - \max_{i \in \text{pa}(i)} t_j) \cdot \mathbf{I}(t_i > \max_{i \in \text{pa}(i)} t_j) \tag{4}$$

Thus, the set of waiting times $\{T_i\}$ forms CBN with a partial temporal order $T_j < T_i$ for all $j \in pa(i),\ i = 1, 2, \ldots, N.$.

Combining Eq. (2) with Eq. (3), we obtain that the probability of the genotype X is

$$\text{Prob}_{\lambda,P}[X] = \text{Prob}_{\lambda,P}\left[\max_{i:X_i=1} T_i < T_s < \min_{j:X_j=0} T_j\right] \tag{5}$$

It can be calculated by summing over all possible paths starting from zero mutations and leading to the genotype X under the constraints of poset. Beerenwinkel et al. (Beerenwinkel et al., 2007;Gerstung et al., 2009) then developed a Bayesian inference framework handling the experimental errors on the genotype, e.g. the vector X could contain false positive (or also false negative because they are below the limit of detection). To manage this situation, Beerenwinkel et al. introduced the observed genotype $Y = \{Y_1, Y_2, \ldots, Y_n\}$, and a probability that a mutation is falsely observed. From now on, X will be called the "true" genotype, while Y will be called the observed "genotype". Since in this model the conditional variables $Y_i|X_i$ are independent for each $i = 1, 2, \ldots, N$, the conditional probability of an observation Y given the genotype X is

$$\text{Prob}[Y|X] = \prod_{i=1}^{n} \text{Prob}_\epsilon[Y_i|X_i] = e^{d(X,Y)}(1-\epsilon)^{n-d(X,Y)} \tag{6}$$

where $d(X,Y) = \sum_{i=1}^{n} |X_i - Y_i|$ is the Hamming distance. Using the Bayes theorem, where the probability of the "true" plays the role of the prior probability, the posterior probability of obtaining the genotype X given an observation Y is

$$\text{Prob}_{\epsilon,\lambda,P}[X|Y] = \frac{\text{Prob}_{\lambda,P}[X] \cdot \text{Prob}_\epsilon[Y|X]}{\sum_{X\in G} \text{Prob}_{\lambda,P}[X] \cdot \text{Prob}_\epsilon[Y|X]} \tag{7}$$

where G is the graph representing the occurrence of mutations of the genotype compatible the poset P. The denominator of Eq. (7) is the marginal likelihood of the observed data (also known as "evidence"), i.e. $\text{Prob}_{\lambda,P}[Y]$. Beerenwinkel et al. (Beerenwinkel et al., 2007;Gerstung et al., 2011) showed how the parameters λ can be estimated by an EM algorithm, while the poset can be estimated by a simulated annealing procedure.

1.3 Evolutionary algorithms

Evolutionary computation was proposed about 30 year ago and is now a re-emerging approach to model the accumulation of mutations and their interdependencies. Evolutionary computation groups a set of techniques – genetic algorithms, evolutionary programming, genetic programming and evolutionary strategies – being inspired to the mechanics of natural selection and natural evolution. Evolutionary algorithms simulate the natural evolution by searching and finally selecting the individuals, within a given population, that are more likely to survive. The quality of an individual, and thus its survival probability, is measured by an objective function known as *fitness*. The outputs of the evolutionary algorithms are (i) the set of individuals reporting high scoring fitness, and (ii) the evolutionary trajectories toward the population of such individual.

The use of evolutionary algorithms in reconstructing the temporal sequence of genetic alterations leading to cancer is based on the assumption that the evolutionary dynamics of cancer mimics the principles of population genetics. Traditionally, law and dynamics of population genetics are

modelled as stochastic evolutionary processes. For instance, consider a population of N cells at risk of accumulating genetic mutations responsible for cancer. Cell proliferation is assumed to be a stochastic process, according to which, at each time step a cell is chosen proportional to its fitness to produce a mutated daughter cell. A mutated cell can take over the population, or dies due to stochastic fluctuations. Depending on the order of appearance of particular mutations, the population of cells follow different evolutionary paths toward cancer.

A computational approach based on these models of cancer evolution has been proposed by Attolini et al. (Attolini et al., 2010) The approach, called RESIC (Retracing and Evolutionary Steps In Cancer) is used to deduce temporal sequence of genetic events during tumorigenesis. RESIC considers an initial population of N cells with genotype i and fitness r_i equal to the growth rate. r. The cell proliferation is modelled as stochastic process in which a cell is chosen in proportion to its fitness to divide. Each division results in two daughter cells, one of which replaces the original cell and the other replaces a randomly chosen cell. This kind of birth-and-death process is known as Moran process and belongs to the class of Markov processes. During each division, one of the daughter cells may accumulate a mutation.

Assume that the mutated daughter cell has a genotype j and a fitness r_j. Three cases are possible

- $r_j > r_i$, the mutated cell is advantageous as compared to the mother cells
- $r_j < r_i$, the mutated cell is disadvantageous
- $r_j = r_i$, it is selectively neutral.

The probability that a mutated cell reach fixation is defined as follows

$$p(r_i, r_j) = \frac{1-\frac{1}{r_j/r_i}}{1-\left(\frac{1}{r_j/r_i}\right)^N}.$$

A probability u, that during each cell division, a mutation arises, is then introduced in order to define the transition rate between two mutations i and j as follows

$$\rho_{ij} = Nu \cdot p(r_i, r_j)$$

Depending on the values of transition rates, which in turn depend on the fitness values (r_i, r_j), each path in the network of mutations may have a different likelihood to occur. Due to the Markovian assumption, this likelihood is calculated as the joint probability of independent mutation events belonging to that path.

This model is then used to describe the dynamics of populations of patients, each harbouring a population of cells at risk of accumulating mutations. Next to the networks of mutations, RESIC considers a network whose nodes represent samples of patients populating the mutational states indicated in the network of mutations. The scaling from the micro-model (on the level of a population of cells within a single patient) to the macro-model (on the level of a population of patients, each with a population of cells proliferating according to this stochastic process) is obtained by multiplying the transition rates between mutational states within one patient by the number of patients in each mutational state.

Finally, an optimization algorithm is used to estimate the transition rates for which the number of patients in each node coincides with the observation in a cross-sectional dataset. Once the optimized parameters have been estimated, they are used to calculate the probability of each path in the network and thus to identify the most likely.

2 Building a gold-standard: an example

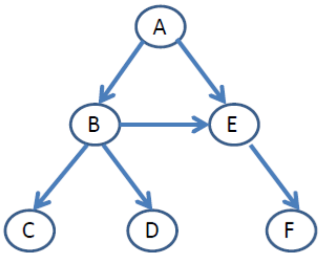

Extract all the paths in the APG starting from the root.

$$P(C|B\cap A) = \frac{P(C\cap B\cap A)}{P(B\cap A)} > 0$$
$$P(D|B\cap A) = \frac{P(D\cap B\cap A)}{P(B\cap A)} > 0$$
$$P(E|A\cap B) = \frac{P(E\cap A\cap B)}{P(A\cap B)} > 0$$
$$P(F|A\cap E) = \frac{P(F\cap E\cap A)}{P(\ A\cap E)} > 0$$
$$P(F|A\cap E\cap E\cap B) = \frac{P(F\cap A\cap E\cap E\cap B)}{P(A\cap E\cap E\cap B)} > 0$$

Assign arbitrarly values to the joint probabilities of the events along each path.

$P(C\cap B\cap A) \approx 10\%$
$P(D\cap B\cap A) \approx 5\%$
$P(E\cap A\cap B) \approx 30\%$
$P(F\cap E\cap A) \approx 50\%$
$P(F\cap E\cap A\cap B) \approx 20\%$

Constraint 1: assign an arbitrarily low value to the conditional probability of disconneted sister events.

$$P(C|D) < \epsilon_1 \quad \text{and} \quad P(D|C) < \epsilon_2$$
$$P(D|F) < \epsilon_3 \quad \text{and} \quad P(F|D) < \epsilon_4, \quad \epsilon_i \ll 1, \quad (i = 1,2,3,4)$$

Constraint 2: assign an arbitrarily low value to the conditional probabilities for the edgees having an opposite direction to the one defiend in the topology.

$$P(B|D) \ll \epsilon_5, \quad P(B|C) \ll \epsilon_6 \quad P(B|A) \ll \epsilon_7, \quad P(B|E) \ll \epsilon_8$$
$$P(E|F) \ll \epsilon_9, \quad P(A|E) \ll \epsilon_9, \quad where \epsilon_j \ll \quad (j = 5,6,7,8,9)$$
$$P(C|A), P(F|B), \text{ and } P(F|A) \text{ no values can be imposed a priori}$$

References

Attolini, C.S., Cheng, Y.K., Beroukhim, R., Getz, G., Abdel-Wahab, O., Levine, R.L., Mellinghoff, I.K., and Michor, F. (2010). A mathematical framework to determine the temporal sequence of somatic genetic events in cancer. *Proc Natl Acad Sci U S A* 107**,** 17604-17609. doi: 10.1073/pnas.1009117107.

Beerenwinkel, N., Eriksson, N., and Sturmfels, B. (2007). Conjunctive Bayesian networks. *Bernoulli* 13**,** 893-909.

Beerenwinkel, N., Rahnenfuhrer, J., Kaiser, R., Hoffmann, D., Selbig, J., and Lengauer, T. (2005). Mtreemix: a software package for learning and using mixture models of mutagenetic trees. *Bioinformatics* 21**,** 2106-2107. doi: 10.1093/bioinformatics/bti274.

Cheng, Y.K., Beroukhim, R., Levine, R.L., Mellinghoff, I.K., Holland, E.C., and Michor, F. (2012). A mathematical methodology for determining the temporal order of pathway alterations arising during gliomagenesis. *PLoS Comput Biol* 8**,** e1002337. doi: 10.1371/journal.pcbi.1002337.
Gerstung, M., Baudis, M., Moch, H., and Beerenwinkel, N. (2009). Quantifying cancer progression with conjunctive Bayesian networks. *Bioinformatics* 25**,** 2809-2815. doi: 10.1093/bioinformatics/btp505.
Gerstung, M., Eriksson, N., Lin, J., Vogelstein, B., and Beerenwinkel, N. (2011). The temporal order of genetic and pathway alterations in tumorigenesis. *PLoS One* 6**,** e27136. doi: 10.1371/journal.pone.0027136.
Hainke, K., Rahnenfuhrer, J., and Fried, R. (2012). Cumulative disease progression models for cross-sectional data: a review and comparison. *Biom J* 54**,** 617-640. doi: 10.1002/bimj.201100186.
Szabo, A., and Boucher, K. (2002). Estimating an oncogenetic tree when false negative and positives

are present. *Mathematical Biosciences* 176**,** 219-236. doi: 10.1016/S0025-5564(02)00086-X.

3 Supplementary Figures

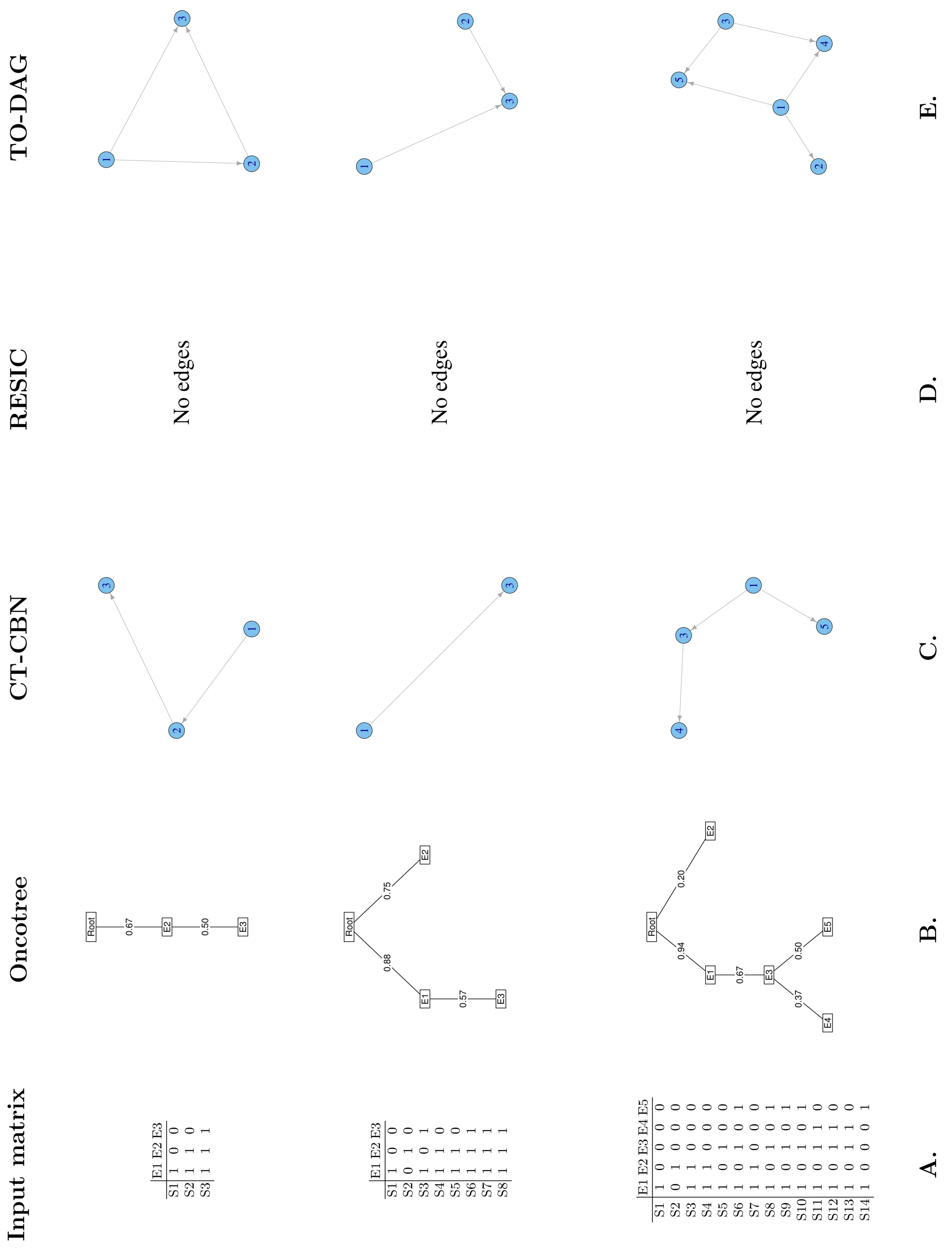

Figure S1: examples of output graphs obtained from co-occurrence tables. **(A)** Columns represent genetic events and rows represent patients. Presence and absence of a mutation is indicated with 1 and 0, respectively. **(B)** Oncotree refers to Oncogenetic Trees methods (Szabo and Boucher, 2002), **(C)** CT-CBN is acronym of Continuous Time Conjunctive Bayesian Network (Gerstung et al., 2011), **(D)** RESIC is acronym of Retracing the Evolutionary Steps in Cancer (Cheng et al., 2012) and **(E)** TO-DAG is the acronym Timed-Oncogenetic Directed Acyclic Graph.

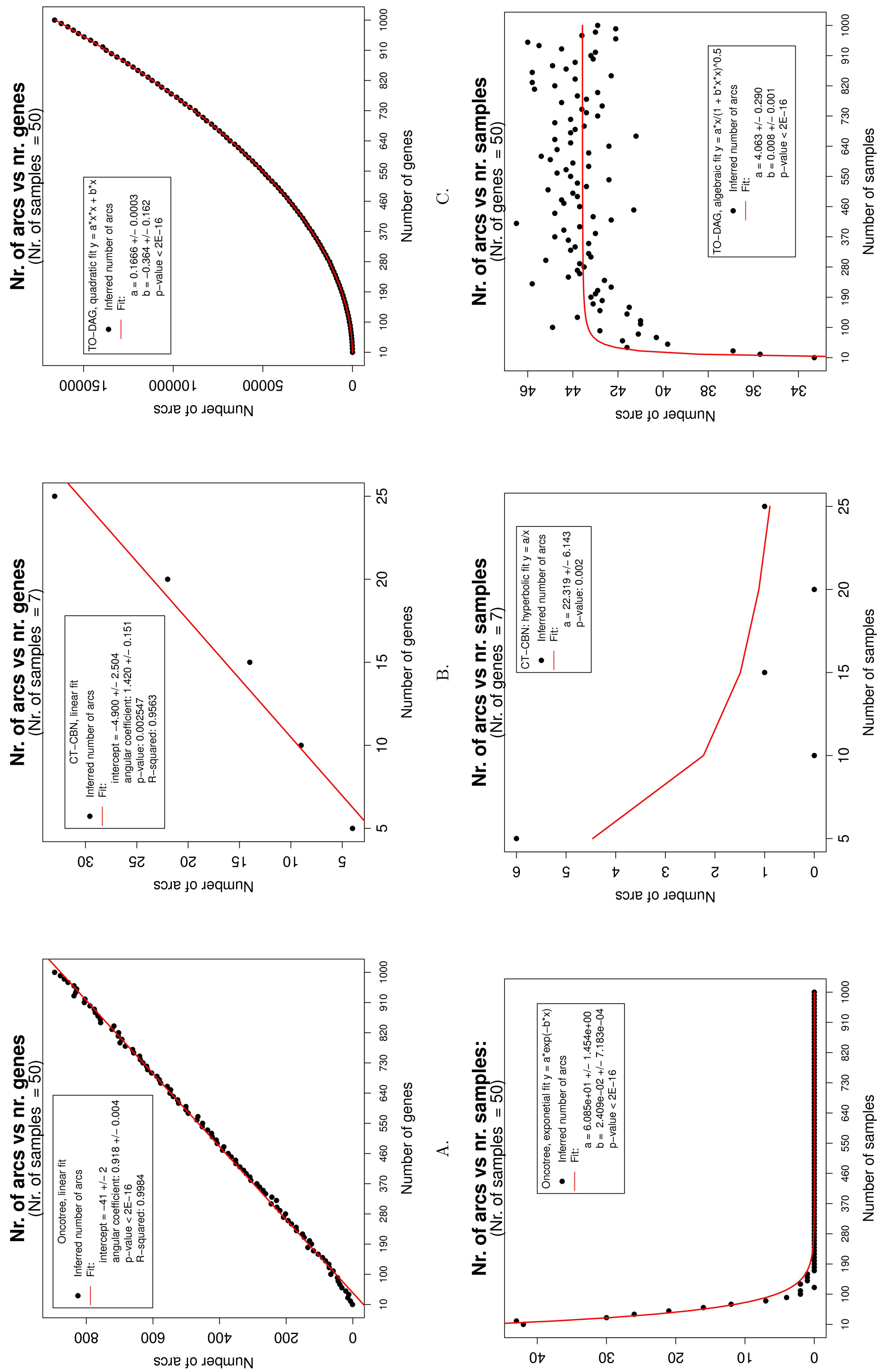

Figure S2: tests on random data for the estimation of predicted number of edges versus number of genes **(A, B, C)** and number of samples **(D, E, F)** for oncotree, CT-CBN graphs and TO-DAG respectively. Analytical forms and parameters of the fitting functions are reported in the legend. RESIC predicts no edges on random data.

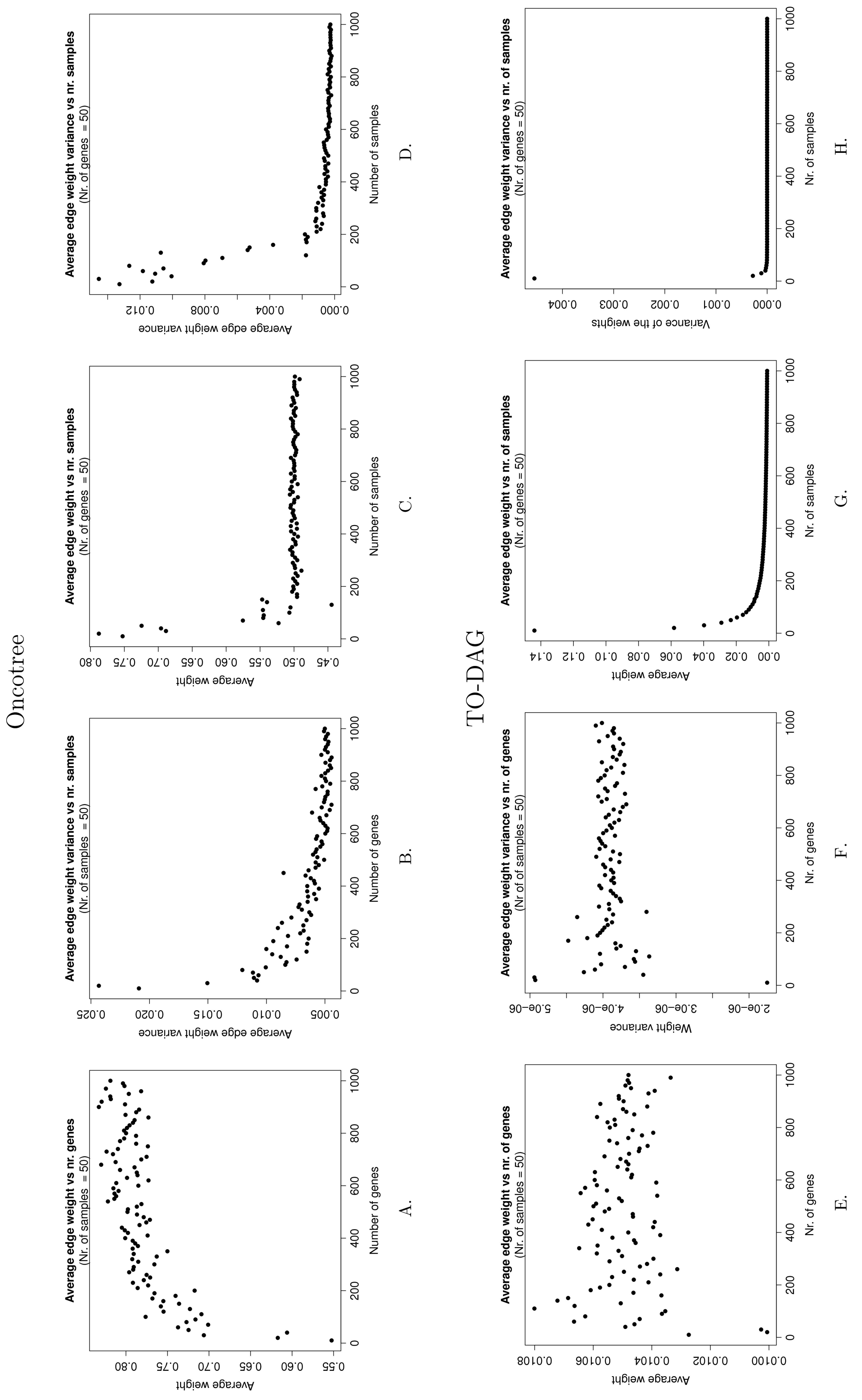

Figure S3: behaviour of average edge weight versus number of genes and number of samples in Oncotree and TO-DAG **(A, E** and **C**, **G**, respectively**)**, and behaviour of average edge weight variance versus number of genes and number of samples in Oncotree and TO-DAG **(B, D,** and **F, H,** respectively**)**. Note that in **(C)** the average edge weight stabilizes around 0.5 exactly when the only existing edges in the tree are those departing from the root, i.e. when all the other nodes are disconnected.

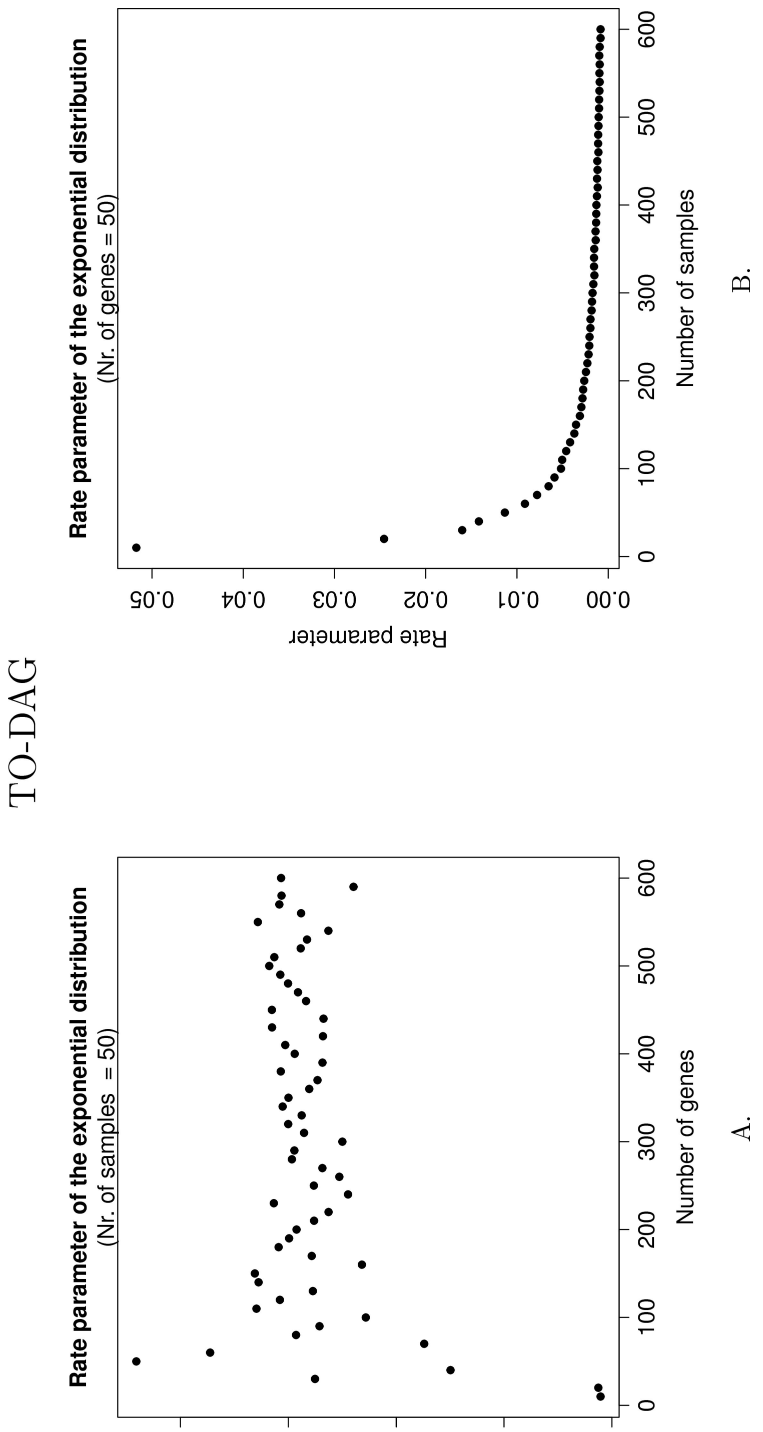

Figure S4: rate parameter of the exponential distribution of the mutation waiting times as function of number of genes **(A)** and number of samples **(B)**.

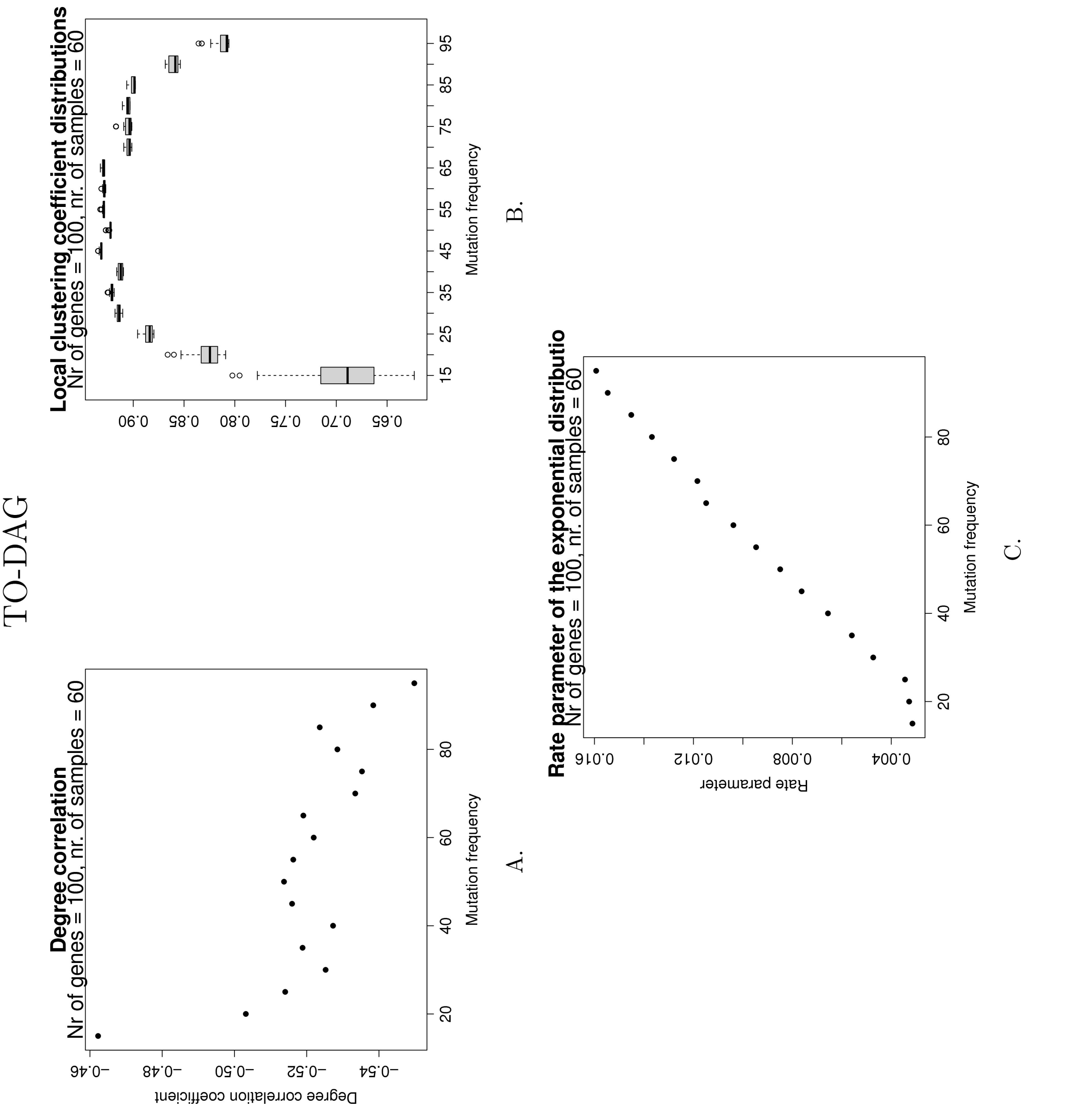

Figure S5: degree correlation coefficient **(A)**, clustering coefficient **(B)** and exponential time distribution rate **(C)** versus mutation frequency.

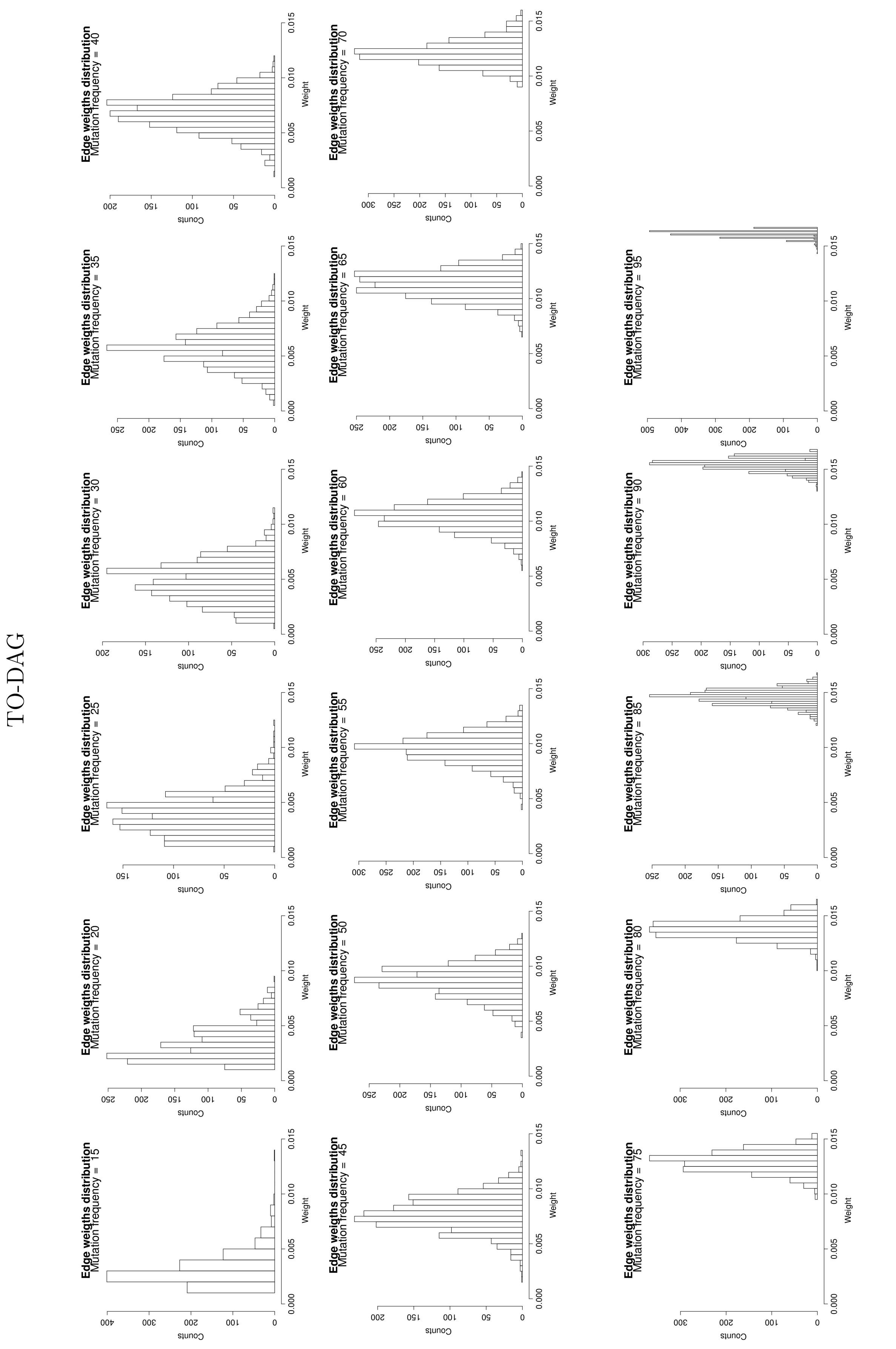

Figure S6: edge weight distribution in TO-DAG model from synthetic data obtained by changing the mutation frequency.

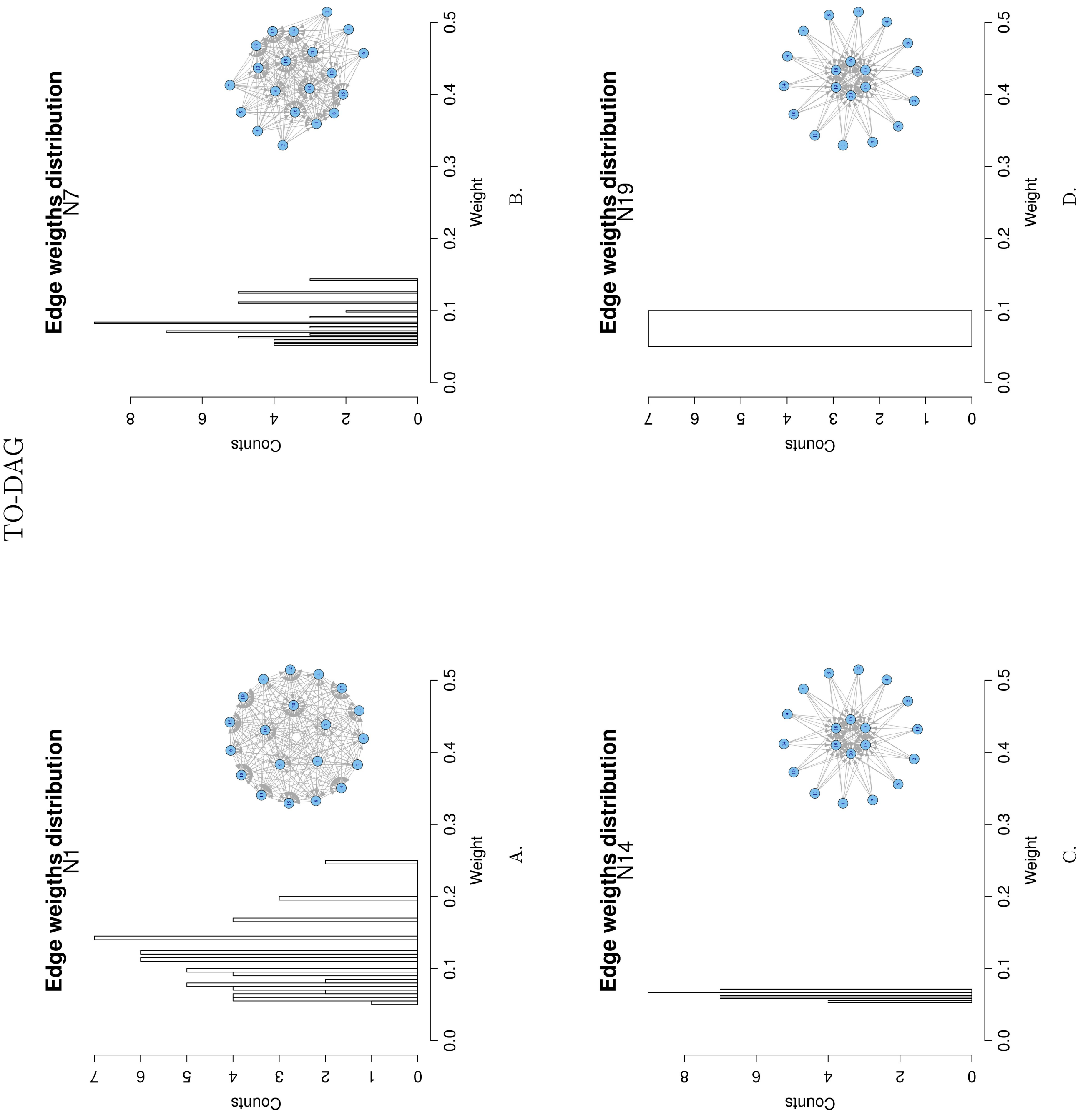

Figure S7: edge weights distributions for the four networks inferred by TO-DAG from synthetic data generated by a step-wise increment of mutation frequency.

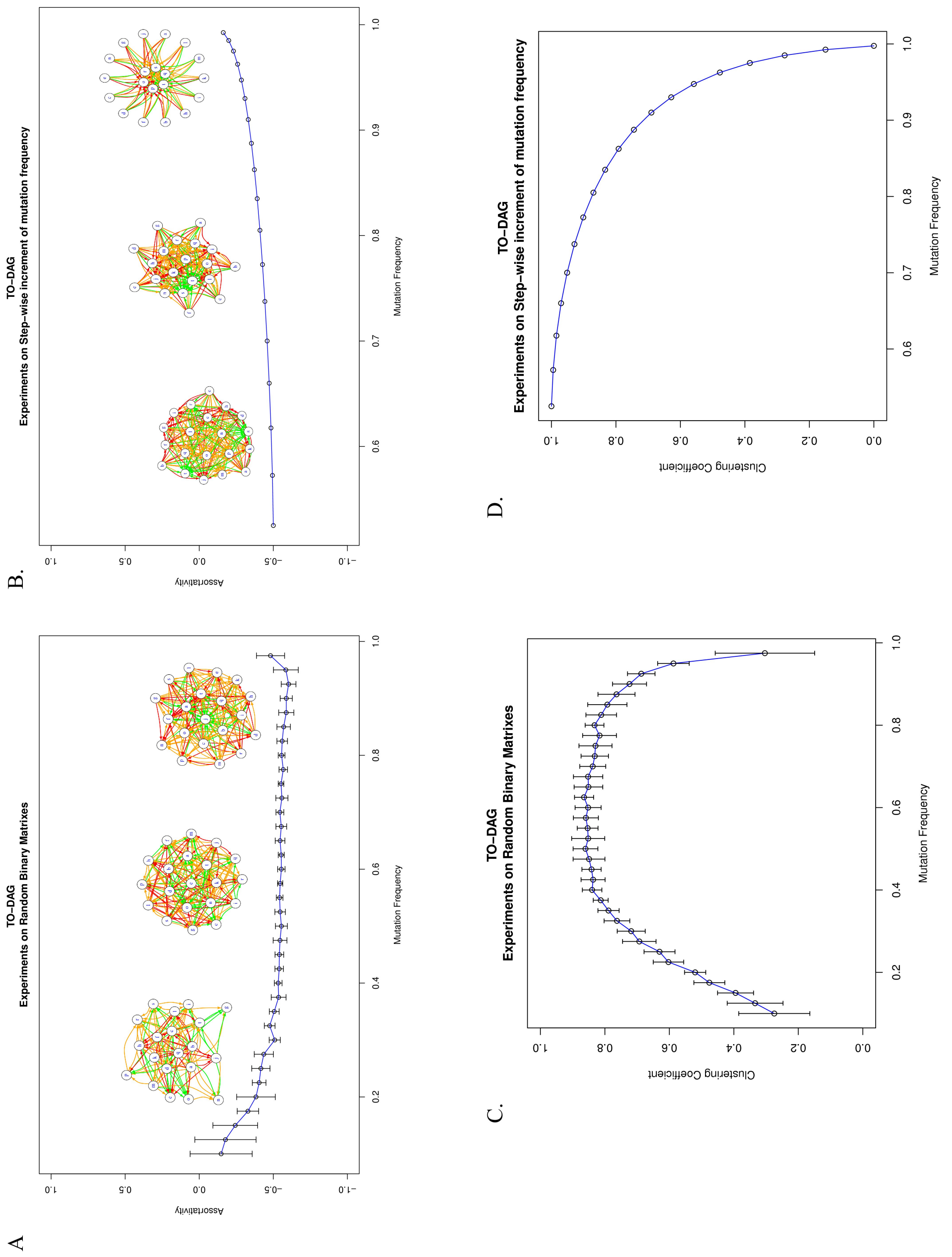

Figure S8: assortativity and topological representations of DAGs in Random and Triangular matrices. **(A, C)** Plots make a comparison between the rate of the assortativity index in random matrix (each point is the averaged value computed over 20 matrices) and triangular matrices. Assortativity values are comparable in the two plots considering mutation frequencies higher than 0.5 and in both cases the index assumes only negative values. Random matrices with lower mutation frequencies have greater mean assortativity values but also greater standard deviations. A plateau-like behaviour is observed in random matrices with mutation frequencies in range 0.4 - 0.8 and a slight increase is observed at highest frequencies. Assortativity index tends to zero when the mutation frequency is getting higher. In each plot, three graphical representations of directed acyclic graphs inferred by TO-DAG are plotted over the assortativity rate at corresponding frequency of mutation to refer to the corresponding structure of the network. **(B, D)** The behavior of transitivity is showed in the two sets of experiments.

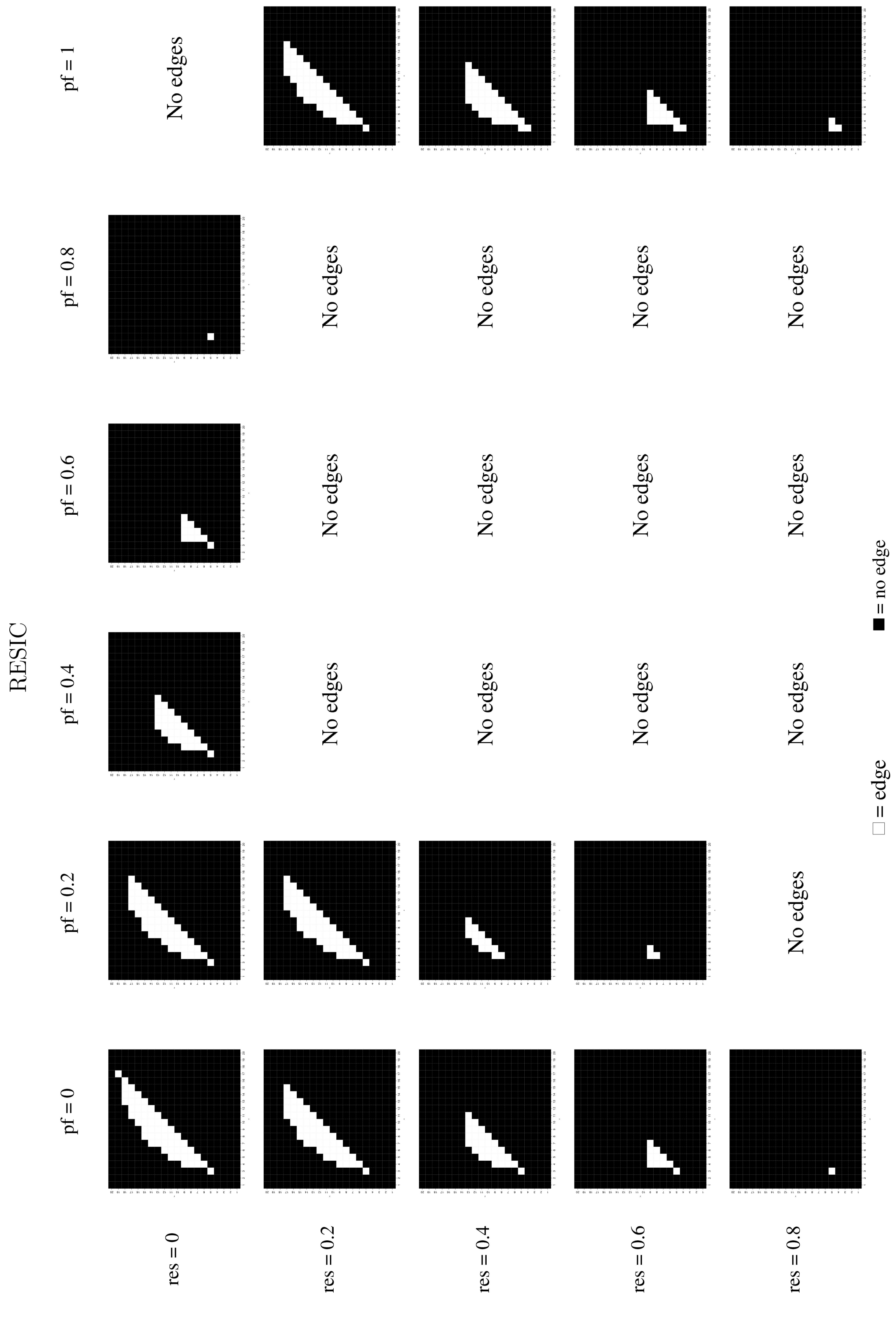

Figure S9: output of RESIC obtained from the binary lower-triangular matrix for different combinations of the parameters “pairfreq” (pf) and “res”. Pairfreq is the minimum co-occurrence frequency of mutations, and “res” is minimum marginal mutation frequency to consider. No edges are predicted. If res is equal to one, irrespectively to the value of pf, no edges are predicted.

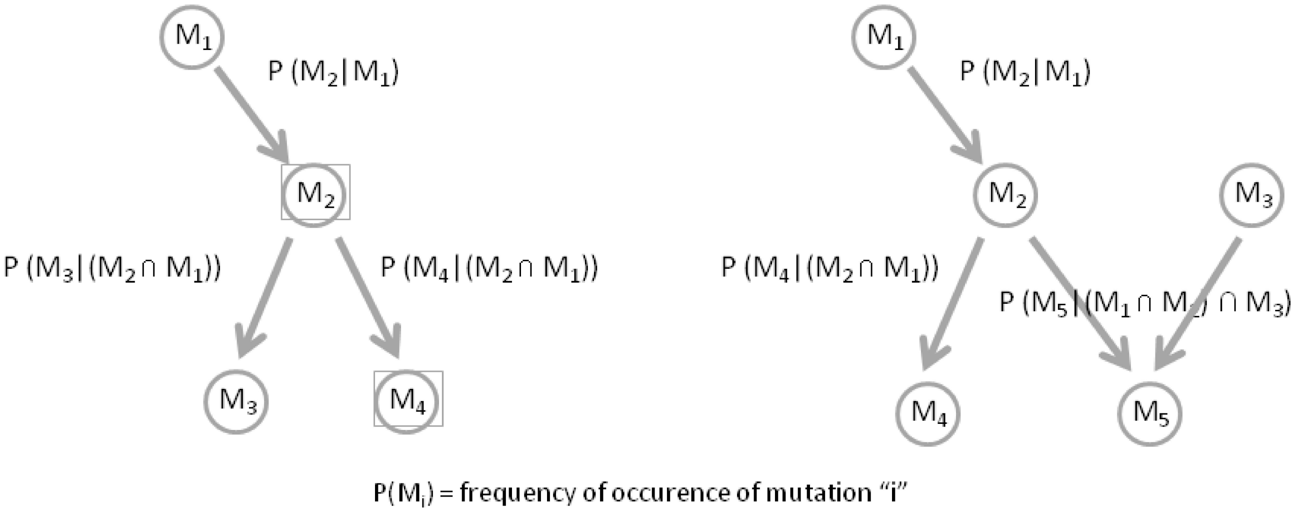

Figure S10: cumulative cancer progression is modelled as a directed acyclic graph representing the causal relationships among mutation events. The occurrence of a mutation in a pattern is conditionally dependent on the occurrence of all the mutations preceding it.

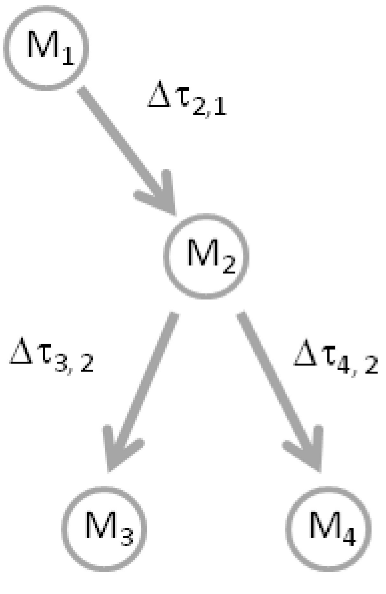

$$\Delta\tau_{j,i} \sim P(M_j|M_i) \cdot \exp(-P(M_j|M_i)\Delta\tau_{j,i})$$

Figure S11: waiting time estimations between genetic events. If M_j is conditionally dependent on M_i, the waiting time for M_j given that that M_i has occurred is a realization on a negative exponential distribution parametrized by the conditional probability of $M_j|M_i$. This model of waiting time does not imply a Markovian character of the process of mutation accumulation, because the conditional probabilities are not defined as function of time.
